# Major histocompatibility complex genes partly explain early survival in house sparrows

**DOI:** 10.1038/s41598-017-06631-z

**Published:** 2017-07-26

**Authors:** B. Lukasch, H. Westerdahl, M. Strandh, F. Knauer, H. Winkler, Y. Moodley, H. Hoi

**Affiliations:** 1 Konrad Lorenz Institute of Ethology, Department of Integrative Biology and Evolution, University of Veterinary Medicine, Vienna; Savoyenstraße 1a, A-1160 Vienna, Austria; 2 Molecular Ecology & Evolution Lab, Department of Biology, Lund University, Ecology Building, Sölvegatan 37, SE-223 62 Lund, Sweden; 3 Research Institute of Wildlife Ecology, Department of Integrative Biology and Evolution, University of Veterinary Medicine, Vienna; Savoyenstraße 1a, A-1160 Vienna, Austria; 4 Present Address: Department of Zoology, University of Venda, Private Bag X5050, Thohoyandou, 0950 Republic of South Africa

## Abstract

Environmental factors and genetic incompatibilities between parents have been suggested as important determinants for embryonic mortality and survival. The genetic set-up of the immune system, specifically the highly polymorphic major histocompatibility complex (MHC) may also influence individual resistance to infections. MHC proteins are important for an appropriate adaptive immune response and enable T-cells to separate ‘self’ from ‘non-self’. Here we investigate the importance of MHC functional diversity for early development in birds, more specifically, if offspring survival and body mass or size depends on number of different functional MHC alleles, specific functional MHC alleles or similarity of MHC alleles in the parents. Unhatched eggs are common in clutches of many bird species. In house sparrows (*Passer domesticus*), embryo and nestling mortality can exceed 50%. To control for environmental factors, our study was carried out on an aviary population. We found that one specific functional MHC allele was associated with reduced nestling survival, which was additionally supported by lower body mass and a smaller tarsus when nestlings have been 6 days old. Another allele was positively associated with tarsus length at a later nestling stage (nestlings 12 days old). These results indicate that MHC alleles might influence pathogen resistance or susceptibility.

## Introduction

Survival and growth rates of young nestlings are known to be directly influenced by environmental factors, such as pollutants, nutrition and microbial infections or indirectly e.g., via stressors or maternal condition^1–5^. Embryonic mortality is sometimes also a result of genetic incompatibility between parents. Incompatibilities may result from in- or outbreeding e.g., refs 2, 6–10. For example, in great reed warblers (*Acrocephalus arundinaceus*) it was found that hatching success of eggs was lower when pairs were genetically more similar^11^. In vertebrates, individual variation in resistance or susceptibility to infections could depend on the genetic set-up of the immune system, where the major histocompatibility complex (MHC) plays a central role. The MHC is a highly polymorphic gene region that encodes proteins that present foreign peptides to T-cells which is central for an appropriate adaptive immune response against pathogens^12^. The amino acids (AA) in the particularly polymorphic peptide-binding regions (PBR) of the MHC molecules determine what antigens can be bound and are therefore crucial for the function of each allele^13^. Potentially, MHC incompatibility between parents leads to embryonic mortality. In human and primate pairs who share MHC alleles, homozygote offspring are underrepresented, possibly because of *in utero* selection against homozygotes^14, 15^.

The highly polymorphic genes at MHC loci are believed to be under some form of balancing selection maintaining the high variation, such as pathogen-driven selection^16^. Pathogen-driven MHC diversity can be maintained in three different, not mutually exclusive ways.

First, individuals that are heterozygote at the MHC loci possess more MHC alleles and are therefore theoretically able to resist a broader range of pathogens compared to homozygotes^17, 18^. On the other hand, having too many different MHC alleles might result in reduced self-tolerance due to negative T-cell selection^19–21^. Theory and data suggest that highest offspring immunocompetence may be achieved at intermediate levels of MHC diversity (i.e. the optimizing hypothesis)^18^. Indeed, some empirical studies in fish and reptiles found the lowest effect of parasite infections in individuals with intermediate rather than high MHC diversity^22–24^. In sticklebacks, females might be able to optimize MHC allele number in their offspring during mate choice by allele counting^25, 26^. MHC dependent mate preference also seems to be important in house sparrows. In a population of house sparrows^27^, found that females with a low number of alleles were most attracted to males carrying a high number of MHC class I alleles. This might reflect a mating-up preference by allele counting to obtain offspring with an optimal MHC diversity.

Second, there is also variation in pathogenic selection pressure in space and time, leading to different subsets of MHC alleles across subpopulations as proposed by the ‘*fluctuating selection hypothesis*’^28^.

Third, according to the ‘*frequency-dependent selection hypothesis*’, especially rare MHC alleles are beneficial in the host population^29^. If they provide a stronger immune response to a specific antigen they can gradually increase in frequency in a population, but once a rare allele becomes common, the parasite will adapt to it and the formerly beneficial allele will no longer be advantageous and decrease in frequency while other alleles increase, maintaining high diversity^28, 29^. In sheep (*Ovis aries*) common MHC alleles were associated with lower lamb or yearling survival, while a rare MHC allele was associated with higher survival of yearlings^30^. Specific MHC alleles have been associated with higher infection rates, parasite loads and survival in rodents, lemurs and birds^31, 32^. Rivero-de Aguilar *et al*.^33^ found associations between specific MHC class I alleles and increased susceptibility or resistance to Leucocytozoon parasites in a population of blue tits.

In birds it is possible to make detailed observations of some of the factors influencing embryonic mortality since embryonic development is taking place outside the female. It is quite common to find unhatched eggs in clutches of most bird species, which could be due to infertility or embryonic mortality^34^. There are some studies on the negative effects of microorganisms on poultry egg development^35–37^, but little is known about their effect on late embryo mortality in free-living birds^38^ and on growth and mortality of young, especially altricial nestlings^39^.

The overall aim of this study was to examine the relationship between survival and growth of young birds in relation to MHC variation, which is the first study so far on birds.

In particular we investigated if survival and growth in young chicks during the nestling stage depends on the number of different functional MHC alleles/individual or specific functional MHC alleles using house sparrows (*Passer domesticus*) as the model system. In house sparrows, mortality of embryos and nestlings can exceed 50%^40^. We predict that survival is highest with intermediate similarity of functional MHC alleles in parents, an intermediate number of different functional MHC alleles/individual in chicks and also specific functional MHC alleles might be advantageous or disadvantageous. We further expect that offspring with an intermediate number of different functional MHC alleles/individual or specific functional MHC alleles may have advantages/disadvantages in terms of growth. Our aviary population of wild house sparrows allowed us to control for several environmental factors and we also had the possibility to collect embryos and dead chicks to obtain DNA samples.

## Results

### MHC data; 454 read numbers and number of functional alleles (FA)

We observed a total of 75894 sequence reads of 19200 unique sequences in R1 that had complete tags and primers. In R2 we observed a total of 125752 sequence reads of 34670 unique sequences. After filtering the data, a total of 85 different MHC alleles were found (65 of them were novel; accession numbers and frequencies in Supplementary Table 3, sequence alignment in Supplementary Fig. 3). On average, individuals had 4.7 MHC alleles (sd: 1.5 (R1) and 1.4 (R2)) ranging from 2–8 (R1) and 1–8 (R2) alleles/individual. These could be translated into 78 amino acid (AA) MHC alleles and 59 unique functional MHC alleles (Supplementary Table 4, Supplementary Fig. 4), based on their chemical properties in the PBR^41^. The average number of functional MHC alleles (FA) per individual was 4.2 (ranging from 1–8). Ten functional MHC alleles occurred at frequencies higher than 10% at the population level, and these were included in the analysis (Supplementary Fig. 5).

### Influence of MHC on survival

DAY 1 (hatching day): Out of 293 fertilized eggs, 31 embryos did not hatch. Embryonic mortality was significantly correlated with clutch order (RVI = 0.76, p = 0.032) (Table 1a). Individuals that were born in later clutches were less likely to survive. The remaining variables only had minor effects (RVI < 0.7 and p-value > 0.05).

**Table 1:** Factors influencing survival.

	Estimate	Adjusted SE	p value	RVI value
**a) 1 day old chicks**				
(Intercept)	0.0033	0.0771	0.9661	
Clutch order	−0.0641	0.0299	0.0322*	0.76
FA25259	−0.1430	0.0866	0.0987.	0.56
FA21013	−0.1028	0.0640	0.1083	0.54
**b) 6 day old chicks**				
(Intercept)	0.4474	0.8277	0.5888	
FA25259	−0.2470	0.1168	0.0344*	0.75
Microsatellite heterozygosity	−1.3630	0.7077	0.054.	0.67
Sex	0.2564	0.1025	0.0124*	0.88
Proportion of FA shared	−0.4575	0.1964	0.0198*	0.83
**c) 12 day old chicks**				
(Intercept)	0.2293	0.2521	0.3631	
Clutch size	−0.08991	0.0351	0.0105 *	0.89
FA25259	−0.2264	0.1236	0.0670.	0.62
Functional MHC distance	−0.2480	0.1415	0.1187	0.59
Sex	0.1412	0.0905	0.1187	0.51

**Figure 1:**
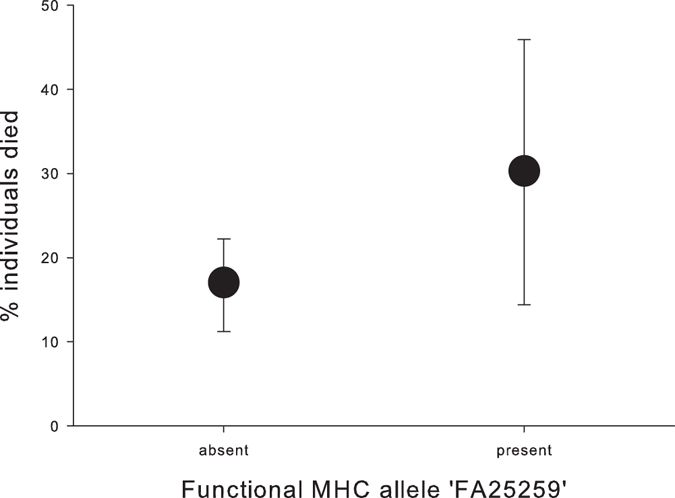
Relationship between the functional MHC allele ‘FA25259’ and nestling mortality at 6 days of age. Presented is the proportion (±binominal confidence intervals) of nestlings that died with this allele being absent or present.

DAY 6 (mid nestling stage): Out of 262 hatched nestlings, 49 died before day 6 and from those, we excluded 11 chicks from the analysis. Since these nestlings did not die naturally (probably killed by neighbouring adults) we are not able to prospect their future survival. Survival was related to the proportion of shared FA in parents (RVI = 0.83, p = 0.020), sex (RVI = 0.88, p = 0.012) and the functional MHC allele ‘FA25259’ (RVI = 0.75, p = 0.034, Fig. 1) (Table 1b). Male nestlings, individuals whose parents shared more functional MHC alleles and individuals with the FA25259 were less likely to survive. The remaining variables only had minor effects (RVI < 0.7 and p-value > 0.05).

DAY 12 (late nestling stage): Out of 213 nestlings that were alive on day 6, 25 died before day 12. Here survival was related to clutch size (RVI = 0.89, p = 0.011) (Table 1c). Individuals that were born in larger clutches were less likely to survive. The remaining variables only had minor effects (RVI < 0.7 and p-value > 0.05).

### Influence of MHC on growth

**Figure 2:**
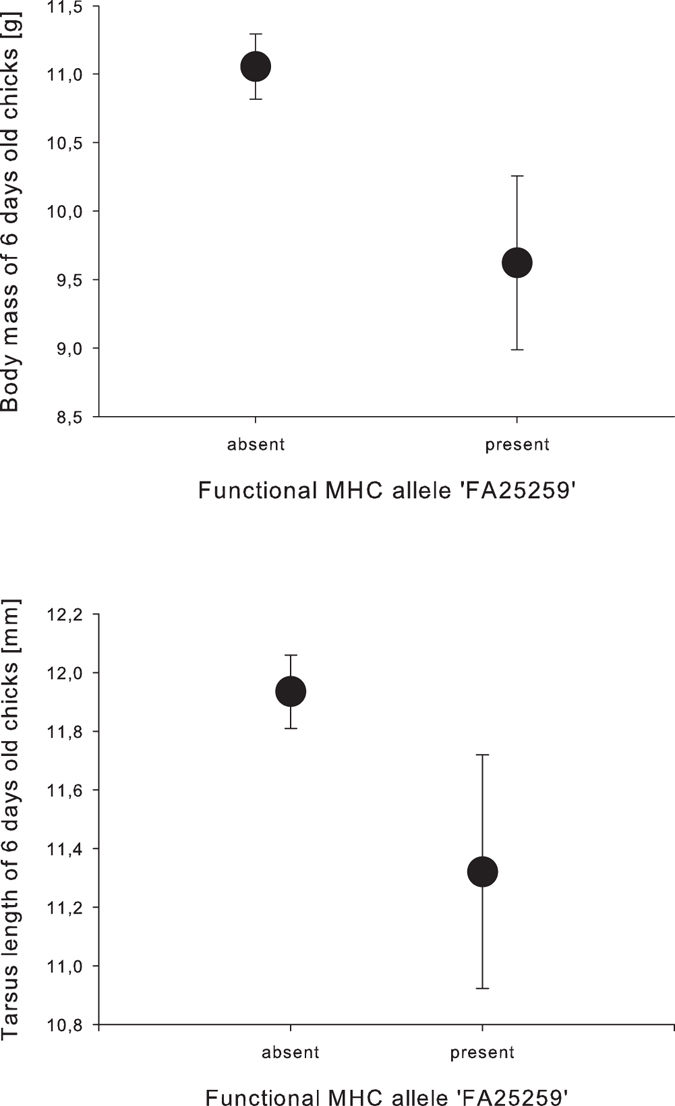
Relationship between the functional MHC allele ‘FA25259’ and nestling body mass and tarsus length at 6 days of age. Presented is the mean ± s.e. body mass in g (upper graph) and tarsus length (lower graph) of 6 days old nestling house sparrows with the allele being absent or present.

DAY 6: In nestlings that were 6 days old (N = 213), body mass was related to clutch size (RVI = 0.91, p = 0.008) and the functional MHC allele ‘FA25259’ (RVI = 0.89, p = 0.012, Fig. 2) (Table 2a). Nestlings with the FA25259 and that were born in larger clutches had lower body mass. Tarsus length on day 6 was related to clutch order (R = 0.92, p = 0.002) and the functional MHC allele ‘FA25259’ (RVI = 0.71, p = 0.048, Fig. 2) (Table 2b) and nestlings with the FA25259 that were born in later clutches had a shorter tarsus. The remaining variables only had minor effects (RVI < 0.7 and p-value > 0.05).

**Table 2:** Factors influencing growth.

	Estimate	Adjusted SE	p value	RVI value
**a) body mass in 6 day old chicks**				
(Intercept)	14.4603	1.8171	<0.0001***	
Clutch size	−0.8707	0.3295	0.0082**	0.91
FA25259	−1.5669	0.6244	0.0121*	0.89
**b) tarsus length in 6 day old chicks**				
(Intercept)	12.2856	0.4752	<0.0001***	
Clutch order	−0.4764	0.1557	0.0022**	0.92
FA00217	0.5216	0.2764	0.0592.	0.64
FA18621	0.5922	0.3538	0.0941.	0.58
FA25259	−0.6697	0.3391	0.0483*	0.71
**c) body mass in 12 day old chicks**				
(Intercept)	24.0188	1.1005	<0.0001***	
Clutch order	−1.4329	0.4573	0.0017**	0.97
Sex	−0.9472	0.5067	0.0616.	0.66
**d) tarsus length in 12 days old chicks**				
(Intercept)	17.997	0.3439	<0.0001***	
Clutch order	−0.4297	0.1457	0.0032**	0.96
FA18621	0.6831	0.2942	0.0202*	0.82
Sex	−0.3177	0.1670	0.0571.	0.67

**Figure 3:**
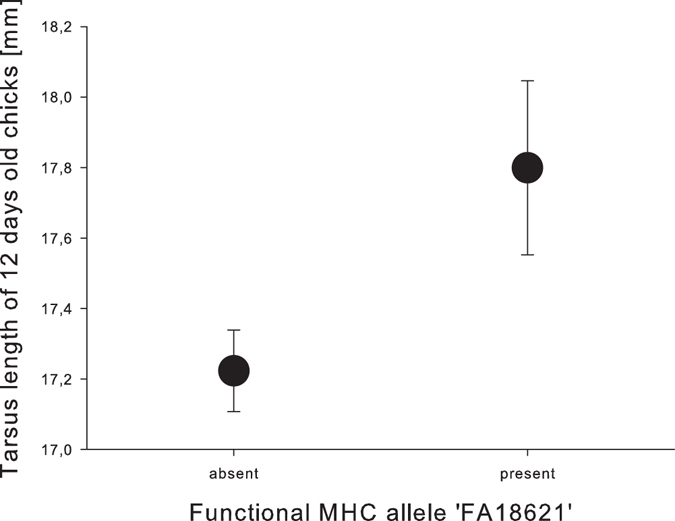
Relationship between the functional MHC allele ‘FA18621’ and nestling tarsus length at a later nestling stage. Presented is the mean ± s.e. of tarsus length in mm of 6 days old nestling house sparrows with the allele being absent or present.

DAY 12: In nestlings that were 12 days old (N = 188), body mass was related to clutch order (RVI = 0.97, p = 0.002) (Table 2c) and nestlings that were born in a later clutch were lighter. We also found that tarsus length on day 12 was related to clutch order (RVI = 0.96, p = 0.003) and the functional MHC allele ‘FA18621’ (RVI = 0.82. p = 0.020, Fig. 3) (Table 2d). Nestlings that were born in an earlier clutch and possessed the FA18621 had a larger tarsus. The remaining variables only had minor effects (RVI < 0.7 and p-value > 0.05).

## Discussion

Our results revealed that early survival of nestlings and growth was associated with two specific functional MHC alleles (FA25259 and FA18621). Individuals with the FA25259 had a reduced survival and body mass and size on day 6, while individuals with the FA18621 had a larger tarsus on a later nestling stage (day 12). FA25259 is encoded by three different MHC alleles, Pado-UA_269, Pado-UA_330 and Pado-UA_339, that translate into three different AA sequences. FA18621 is encoded by Pado-UA_286, Pado-UA_248 and Pado-UA_349 and translate into three AA sequences as well. Supplementary Fig. 6 shows a maximum-likelihood tree with these three important alleles in relation to all house sparrow MHC class I alleles found in this and also other studies. FA25259 occurs in 16.05% of all individuals and in 14.48% of the offspring and seems to be maladaptive in the early developmental period. FA18621 occurs in a frequency of 16.26% in all individuals and in 15.15% of the offspring and seems to have a beneficial effect on body size (growth). Also on day 6, it tended to almost significantly (RVI = 0.58, p = 0.0941) influence tarsus length. We predicted that offspring with an intermediate number of different functional MHC alleles or specific functional MHC alleles may have advantages/disadvantages in terms of survival and growth. Survival and growth on day 6 was lower in individuals that possessed the functional MHC allele FA25259. The importance of this FA is not only reflected in nestling survival on day 6. It was almost significant at the day of hatching (day 1:RVI = 0.56, p = 0.099) and day 12 (RVI = 0.62, p = 0.067) as well, highlighting its importance for survival throughout the whole period in the nest. If this FA had a negative effect on survival, one would expect that nestlings with this FA also suffer lower body condition. As expected, 6 days old nestlings with this FA had a lower body mass and a smaller tarsus. Given that an effect on nestling growth could have been easily masked by unlimited food availability in our aviaries, the influence of this FA seems to be really significant. Unlimited food supply however may be responsible for the non-significant results on growth at a later nestling stage (12 days old nestlings).

MHC class I molecules are important in adaptive immunity, but also young nestlings depend on it. Maternal antibodies are transferred via egg yolk and can influence offspring growth rate by passively protecting them from common pathogens before their endogenous immune system has matured^42^. We found that two specific functional MHC alleles were associated with survival and growth in nestlings. These findings could indicate that these alleles are in linkage disequilibrium with other immune genes, *e*.*g*. MHC class II or genes involved in the innate immune response. While MHC class I molecules typically present peptides derived from the degradation of intracellular pathogens (such as viruses or some protozoa), MHC class II molecules present peptides from extracellular pathogens (such as bacteria)^43^. In order to fully understand the importance of MHC genes in early development, it would be necessary to disentangle the influence of MHC genes itself or any other genes in linkage disequilibrium with it. In our study it is not possible to prove to what extent early mortality was caused by microbial infections, but since survival tended to be negatively correlated with the same functional MHC allele throughout the nestling stage, a microbial infection would be a possible explanation. In this context it would be interesting to link bacterial loads on egg-shells and in nestlings to MHC alleles in parents and/or chicks.

In hole nesting house sparrows, mortality of embryos and nestlings can exceed 50%^40^. Pinowski, Barkowska, Kruszewicz and Gruszewicz^44^ found similar results in a house sparrow population, where egg mortality (including unfertilized eggs and embryonic mortality) was about 30% and nestling mortality ranged between 24 and 30%. 67% of the unhatched eggs were infested with pathogens such as *Escherichia coli* and *Staphylococcus epidermitis*. Also a great deal of nestlings that were infected with pathogenic microorganisms, had a lower growth rate or died, highlighting the negative effects of pathogenic microorganisms on hatching failure, survival and growth. Many factors can influence embryo mortality, including exposure to variable temperatures and humidity, but also microbial infections^4, 5^. These infections could be viral (Adenovirus), bacterial (Salmonella, *Staphylococcus aureus*, *Staphylococcus epidermis*, *Streptococcus faecalis*, *Escherichia coli*, *Mycoplasma infection*, *Chlamydia*) or fungal (*Aspergillus*)^1, 45, 46^.

MHC has become a major study subject of evolutionary and ecological immunology across a large variety of taxa^43, 47, 48^. It has been shown that in some species, especially common MHC alleles are associated with lower survival and higher parasite loads, possibly because escaping the most common host alleles is the primary goal of parasite evolution^30, 31^. In our population, we also found one common functional MHC allele (with a frequency of 16.05%) that had negative effects in early development, while another one (with a frequency of 16.26%) had a positive effect. Previous studies have shown that specific MHC supertypes (clustered according to their antigen-binding motifs) were associated with different components of individual fitness and survival^13, 49, 50^. In great tits, individuals with specific MHC supertypes had higher survival rates and lifetime reproductive success (LRS)^51^. Also in captive-bred prairie-chicken, Bateson *et al*.^52^ found that post release survival as well as immune responses at the time of release were related to specific MHC alleles.

It has been shown that incompatibilities between mates could cause embryonic mortality^14, 15, 53^. We therefore predicted that early survival should be highest with intermediate MHC similarity in parents (tested as the proportion of functional MHC alleles shared and the distance of MHC alleles and functional MHC alleles). We found that the proportion of functional MHC alleles shared in parents was associated with survival on day 6, indicating that individuals were more likely to die if their parents shared more alleles. This could somehow be related to inbreeding effects, since incompatibilities can result from in- or outbreeding, e.g., refs 2, 6–10. In a population of vultures, Agudo *et al*.^54^ found a positive correlation between functional MHC diversity and breeding success.

To control for neutral genetic variation, we also included neutral heterozygosity at 10 polymorphic microsatellite loci in the analysis. As expected, we did not find an association between neutral heterozygosity and survival^55^.

An interesting side result of our investigation is also that clutch order and clutch size seem both important to explain variation in growth and survival. Chicks from earlier clutches were more likely to survive day 1 and had more weight on day 12 and larger tarsi on day 6 and 12. Since we provided the same amount of food during the whole breeding season, one explanation might be that birds allocated more resources to egg production to their first clutch compared to later clutches^56, 57^. Chicks from clutches with fewer eggs had a higher probability to survive on day 12 and had a higher body mass on day 6. Clutch size has been shown to influence juvenile survival and offspring from larger broods are faced to more intense competition for parental care and food e.g., ref. 58.

Also sex was an important predictor of survival of 6 days old birds and females were more likely to survive. Sex-biased mortality of several bird species has already been shown between hatching and fledging^59^.

## Methods

In 2010, we captured wild house sparrows (*Passer domesticus*) in Vienna, Austria. These birds were housed in large outdoor aviaries (10 birds/aviary; aviary size: 3.5 m × 3.5 m × 3 m) at the Konrad Lorenz Institute of Ethology, Vienna. In April 2012, 62 males and 73 females of these birds were put together in mixed flocks (4–5 breeding pairs/aviary) in 14 aviaries that were equipped with nest boxes and nesting material. All aviaries were equipped the same way with vegetation, perches, commercial food for granivorous passerines (ad libitum) and water. Our aviary population of wild house sparrows allowed us to control for several environmental factors that might interfere with our results, like food availability, nest box quality and availability, predation and differences in microclimate as well as socio ecological factors including breeding density and sex ratio.

Data about the start of egg laying, number of clutches/female and clutch size were collected. We analysed 293 offspring (107 females and 186 males also including embryos), that derived from 59 different families (average: 5 chicks/family) and were born in different clutches/female (offspring from clutch 1 (140), 2 (75), 3 (66) and 4 (12)). Survival status was assessed at three different time points, when chicks were 1, 6 and 12 days old. Survival on day 1 reflects hatching success and hatched chicks were tested against unhatched embryos. In cases where we were not sure about a natural cause of death (e.g. chicks thrown out of the nest box), we excluded them from the survival analysis. We used body mass and tarsus length at a given age as an indirect measure of growth. In this context, tarsus length and body mass were measured when chicks were 6 and 12 days old. Body mass was used as a quality indicator because it is assumed to reflect a juvenile’s fat reserve that is also linked to survival^60^. Tarsus length was used as a size indicator, it has been suggested that body size confers an advantage to physically compete for resources against siblings^61^. Data from several passerine studies showed positive correlations between weight, body size and survival at the time of fledging review^62^.

We examined nest boxes and aviaries every day and sampled eggs/embryos/chicks that were thrown out or died in the nest boxes. Five days after the first chick in a clutch hatched, we opened the unhatched eggs (hatching is normally synchronized within one to three days for each clutch). Since it is rather difficult to distinguish between infertility and early embryo mortality, especially if mortality occurred before any visible signs of embryonic development^63^, only eggs that contained a visible embryo where used for the analysis. From a total of 397 eggs, 104 (26.2%) were excluded because no embryo was visible, leaving 293 in the analysis. Tissue from embryos was collected to extract DNA from it. Blood samples (50 µl per bird in total) were collected from the brachial vein in adults and nestlings during the breeding season to extract DNA from it to assess neutral diversity and paternity with microsatellites and MHC diversity. We assessed paternity for all embryos and nestlings and found that 4.1% were sired by extra-pair males. Since these events were very rare and widely distributed throughout families, we did not account for that in the analysis. All observations and measurements were performed prior to MHC screening and therefore blindly with respect to individuals’ genotypes. In Supplementary Table 1 we present a genotype and phenotype table for all the 293 offspring including information about sex, family origin, clutch order and size, body measurements, survival status, microsatellite heterozygosity, number of different MHC alleles and functional MHC alleles/individual, proportion of functional MHC alleles shared in parents, distance of MHC alleles and functional MHC alleles in parents and the presence/absence data of the 10 most common functional MHC alleles. All animal experiments were performed in accordance to the Austrian Law, confirmed and approved by the Federal Ministry of Science, Research and Economy (GZ: BMWF-68.205/0081-ll/3b/2012).

## Molecular Methods

### DNA extraction

Avian blood and tissue samples were stored in 95% ethanol. The ethanol was evaporated and DNA was extracted according to the manufacturer’s instructions using the DNeasy Blood & Tissue Kit (Qiagen).

### MHC genotyping

MHC characterization was carried out using 454 amplicon sequencing. MHC class I exon 3 sequences that encode parts of the peptide binding region in MHC molecules were amplified using individually tagged 454-adapted primers according to ref. 64. The MHC-specific primers used were forward primer (longfw2): GTCTCCACACTGTACAGYGGC; and reverse primer (rv3): TGCGCTCCAGCTCCYTCTGCC^65^. These primers amplify 222–225 bp long (primers not included) classical MHC alleles in house sparrows^65^. Using Qiagen Multiplex MasterMix each 15 µl PCR reaction contained 7.5 µl of Hot Start Master Mix, 0.6 µl (5 µmol) each of the forward and reverse primer, 1 µl of template (25 ng genomic DNA) and 5.3 µl dd H_2_O. The PCR conditions were: 95 °C/15 min, 35 × (95 °C/30 s, 65 °C/1 min, 72 °C/1 min), 60 °C/5 min. PCR products were run on a 2% agarose gel and products with positive amplifications were purified using the MinElute PCR Purification Kit (Qiagen). Samples were pooled in eights and DNA was quantified with a Nanodrop instrument and adjusted accordingly before a second pooling for the 454 quadrants. The number of samples in the 454 quadrants was aimed at a sequence coverage of 300 reads per individual according to ref. 66. 491 individuals were run in two different sequencing reactions (run 1 (R1) and run 2 (R2)) and these runs were filtered separately (see below). R1 contained 272 samples (243 individuals +29 technical replicates (11.9%)) and R2 contained 286 samples (248 individuals +38 technical replicates (15.3%)). 454 sequencing was done at Lund University Sequencing Facility (Faculty of Science), Sweden.

### MHC - bioinformatics and data processing

After 454 sequencing, the data were extracted and assigned to samples using the program jMHC^67^. Raw data from the 454 run were filtered to remove artefacts that were generated during the initial PCR, the emulsion PCR and the 454 sequencing reaction. Filtering steps were in accordance with^51, 64, 68^.

First, sequences in low abundances (<3 reads) were deleted. Identical sequences within individuals were detected and merged using the web-applications ‘seqeqseq’ (http://mbio-serv2.mbioekol.lu.se/apps/seqeqseq.html) and ‘mergeMatrix’ (http://mbio-serv2.mbioekol.lu.se/apps/mergeMatrix.html).

Second, we only retained sequences with a suitable sequence depth of coverage. Since the maximum number of classical MHC alleles in house sparrows is eight, we chose a threshold of 104 reads per sample, thus giving a genotype score of 99.9% m = 8, ^64^. Here we used the web-application ‘popMatrix’ (http://mbio-serv2.mbioekol.lu.se/apps/popMatrix.html) and filter 1 (from a selection of four filters). With filter 1 it is possible to specify a cut-off point for the minimum total sequence abundance/individual. A brief description of all the filters on this web-application can be found in Strandh, *et al*.^69^.

Third, true alleles are likely to occur more frequently per sample than artefact alleles. Therefore we used our replicates to set these thresholds in R1 and R2, deleting all alleles that occurred at lower than 2% per sample (individual) using filter 3.

Fourth, to verify true MHC alleles we determined that they should occur in at least two independent PCRs. However, since we worked with pedigree data (189 adults produced about 300 chicks, individuals were distributed across R1 and R2), every allele is therefore likely to occur several times. Therefore we used the criteria that every allele should be observed at least twice, here we used filter 4.

Fifth, sequences thus obtained were examined in BioEdit (v7.2.0) and those not matching the expected length of 222–225 bp or displaying indels that were not multiples of three base pairs were deleted. Next, PCR recombinants (chimeras) and 1-bp substitutions were removed by examining all sequences starting with those with the lowest population level frequencies^51^. All sequences occurring at a frequency below 2% at the population level were checked by eye, as were 50% of the sequences between a frequency of ≥ 2% to 25%, but no chimeras or 1-bp substitutions were observed. Therefore, we assume that sequences with a frequency of ≥ 2% were true alleles.

The filtered MHC DNA sequences were translated into amino acid (AA) sequences. These sequences were coded according to the chemical binding properties of the AA in the PBR and are hence called “functional MHC alleles” (FA). The 16 AA from each sequence corresponding to the PBR in chicken^70^ were extracted and converted into five physicochemical descriptor variables: *z*1 (hydrophobicity), *z*2 (steric bulk), *z*3 (polarity), *z*4 and *z*5 (electronic effects)^41^.

Distance measures between parents were calculated according to Strandh, *et al*.^69^. The genetic distances of MHC alleles between parents were calculated from a maximum-likelihood tree (2000 bootstraps) that was inferred for all unique AA translated MHC sequences (outgroup MHC class I from chicken, GenBank Acc nr AB159063) using the RAxML software (ver 7.0.4) under the PROTMIX model and the JTT substitution matrix, with default settings (Supplementary Fig. 1). Pairwise overall MHC AA distances between individuals were then computed from this tree with the software Fast UniFrac^71^ available at http://unifrac.colorado.edu. The distances between functional MHC alleles of parents (reflecting the difference in what pathogen antigens can be bound and detected between parents) were assessed by calculating the difference in chemical binding properties of the AA in the PBR. These functional MHC alleles (represented by the *z-*descriptors) were used to construct alternative maximum-likelihood trees with ContML in the PHYLIP-package (v. 3.69). The tree was rooted as previously with the outgroup *Gallus gallus* (AB159063) (Supplementary Fig. 2). This tree represents clusters of functionally rather than evolutionary similar MHC sequences. Pairwise overall distances of functional MHC alleles were computed from this tree in the same way as for the distances of MHC alleles (UniFrac).

MHC diversity was evaluated as: 1) the number of different functional MHC alleles in offspring 2) the proportion of functional alleles shared in parents ( = [100/total number of alleles male + female]*number of shared alleles) 3) distance of MHC alleles in parents 4) distance of functional MHC alleles in parents and 5) the occurrence of the most common functional MHC alleles (>10% at the population level) per individual. We decided to use the 10% percentile as a cut-off point in our frequency distribution. Below this the sample size reaches a critical value with lower statistical explanatory power. Points 2, 3 and 4 are all measures of MHC similarity within parents. With our multilocus data of functional MHC alleles it was not possible to say if a specific allele was homo- or heterozygote, so we use presence/absence data.

### Neutral genetic variation

For the assignment of neutral individual heterozygosity and paternity, we amplified 13 highly polymorphic neutral microsatellite loci and one sexing locus (P2D/P8). Names and origins of the markers are given in Supplementary Table 2. The quality of loci (Hardy-Weinberg Equilibrium and Linkage Disequilibrium) was tested using the programs Cervus (v3.0.3) and Fstat (v2.9.3.2). Individuals were genotyped in two multiplex reactions according to the protocol of Dawson, *et al*.^72^ (Supplementary Table 2). The loci used in this protocol are known to be distributed across at least seven different chromosomes and thus likely reflect the genome-wide level of neutral genetic variation^72^. Forward primers were fluorescently labelled using different dyes (YYE, FAM or AT550, Microsynth). The two multiplex PCRs were carried out using a Multiplex Kit (Qiagen) in a final PCR reaction volume of 6 µl containing 3 µl of the Qiagen enzyme, 2 µl of the primer mix and 1 µl of the template (25 ng genomic DNA). PCR cycling scheme and primer concentrations of the 2 multiplex PCRs were carried out according to Dawson, *et al*.^72^. PCR products were diluted (1:20) and analysed on an ABI genetic analyzer (Applied Biosystem 3130xl). Allele sizes were determined using GeneMapper (v3.0) with reference to an in-house DNA Size Standard (HMROX). However, we excluded locus Pdoµ6 due to amplification problems and Pdo9 and Ase18 because they deviated from the Hardy-Weinberg Equilibrium, this left 10 highly polymorphic neutral microsatellite loci and one sexing locus in the final analysis.

### Statistical analysis

We used generalized linear models within the statistical software R v.3.0.3 x64^73^ to analyse the effects of clutch size, clutch order, sex, microsatellite heterozygosity, number of functional MHC alleles and the presence/absence data of the 10 most common functional MHC alleles on survival on day 1, 6 and 12 and growth on day 6 and 12. Additionally, we included the following variables for survival on day 1, 6 and 12 that corresponded to MHC similarity in parents: Proportion of functional MHC alleles shared in breeding pairs, genetic sequence distance (reflecting the difference in all unique AA) and functional distance (reflecting the difference in the PBR) in breeding pairs. We included these variables because incompatibilities between mates could also cause embryonic mortality^14, 15, 53^. Since early development could have an important influence on growth and survival of chicks, the day they were born (day numbers in 2012) as well as the clutch order (we used birds that originated from clutch 1–4 of specific females) were recorded. Since breeding was highly synchronized, these two parameters were correlated (p < 0.001, R = 0.68) and only clutch number was used. We used the identity link in models with continuous dependent variables (weight and tarsus) and the logit link in the case of dichotomous dependent variables (survival). We included the breeding aviary, the specific clutch and the mother as random effects to avoid pseudo replication. The mother was included because female body condition is an important predictor of breeding success. Griggio and Hoi (2010)^74^ found that in a captive population of house sparrows, females in better condition laid larger clutches and started to breed earlier. We had to expect autocorrelation between the individuals within clutches, between the clutches within the females and between the females within the aviaries. Therefore we used clutch as random effect nested in female and this nested in aviary. In the logistic case the models did not converge and then we aggregated the data on the clutch level by calculating the arithmetic mean of all variables. This is straight forward in metric variables, but in the two categorical variables (survival and sex) the interpretation slightly changes. Survival is now the average survival in the clutch and sex is the sex ratio of the clutch. Even if this change is not much, the resolution is a bit coarser. However, this approach resulted in models with a metric response variable, which easily converged.

### Model selection strategy

From the statistical point of view, this is an observational study with samples sizes ranging from 188 to 293, and 15 to 20 explanatory variables. Obviously, there is a high risk of overfitting. Therefore we use an information-theoretic approach and base our model selection strategy on Akaike Information Criterion corrected for small sample sizes (AICc)^75^. The best would be to compare all possible models, but this would result in up to 2^20^ (~10^6^) potential combination of models, which cannot be computed in reasonable time. Therefore we had to use a simplified procedure: We used a model with the first 10 variables and calculated the relative variable importance (RVI) based on the AICc values (package MuMIn). The RVI gives the probability for each variable being in the best model given these data and this set of candidate models. Then we kept all variables with a RVI > 0.5 in the model (usually two or three variables) and filled up the model with the next variables up to a maximum number of 10 variables. We continued this until we had used all variables (never more than three rounds). We constructed a final model with all variables with a RVI > 0.5 and used this for interpretation. For each variable we report averaged estimate, adjusted standard error, p-value, and RVI. We considered variables with a RVI of bigger than 0.7 as relevant. We believe that this procedure presents a careful way for variables selection for datasets with many variables and is conservative and robust in the results, because thousands of models have been tested and we always found less than 10 relevant variables. See Tables 1 and 2 for the remaining variables of each model.

### Data accessibility

Genbank Acc.nr. of the 65 novel MHC class I exon 3 alleles found in this study: Pado-UA_253, 261–299 and 328–352; Supplementary Table 3). We uploaded following data to https://vetcloud.vetmeduni.ac.at/owncloud/index.php/s/JkilqVZ8OOroET9: 454 sequence data and all information needed to demultiplex the raw data to assign each read to a sample, the microsatellite genotype data, the aligned MHC sequences for all loci and samples, the aligned functional MHC alleles for all loci and samples, the tree files of MHC alleles and functional MHC alleles underlying our phylogenies, the genotype and phenotype data for all individuals and the pairwise distance measures (distance of MHC alleles and functional MHC alleles in parents).

## Conclusions

We found that survival and growth of young nestlings were associated with the MHC, more precisely with two specific MHC alleles. Survival, body mass and tarsus length on day 6 were lower in nestlings that possessed the functional MHC allele ‘FA25259’, while tarsus length was higher on day 12 when individuals possessed the ‘FA185621’. These results indicate that these MHC alleles might be responsible for a higher susceptibility (FA25259) and resistance (FA18621) to specific pathogens during early development. Besides environmental factors that are known to influence early development and survival, we found evidence for genetic factors to be important as well. It would be interesting to know whether and which specific MHC alleles may act at specific developmental stages of an individual. Thus, to further explore the role of MHC alleles or MHC based compatibilities might be an important contribution to understand variation in individual survival and fitness.

## Electronic supplementary material

Supplementary Information
