# Supplementary material for: Major histocompatibility complex genes partly explain early survival in house sparrows
Source: Sci Rep. 2017 Jul 26;7:6571. doi: 10.1038/s41598-017-06631-z (PMC5529587; doi:10.1038/s41598-017-06631-z)
Supplement: Supplementary file 1 — Supplementary Information [file 41598_2017_6631_MOESM1_ESM.pdf]

Supplementary material

Major histocompatibility complex genes partly explain early survival in house sparrows

Lukasch B[1], Westerdahl H[2], Strandh M[2], Knauer F[3], Winkler H[1], Moodley Y[1] & Hoi H[1]

[1]Konrad Lorenz Institute of Ethology, Department of Integrative Biology and Evolution, University of Veterinary Medicine, Vienna; Savoyenstraße 1a, A-1160 Vienna, Austria

[2]Molecular Ecology & Evolution Lab, Department of Biology, Lund University; Ecology Building, Sölvegatan 37, SE-223 62 Lund, Sweden

[3] Research Institute of Wildlife Ecology, Department of Integrative Biology and Evolution, University of Veterinary Medicine, Vienna; Savoyenstraße 1a, A-1160 Vienna, Austria

Supplementary Table 1: Genotype and phenotype table of 293 offspring

Individual	Sex (M=1, F=2)	Family	Clutch order	Clutch size	Body mass (6 days)	Tarsus length (6 days)	Body mass (12 days)	Tarsus length (12 days)	Survival status '	Microsatellite heterozygosity (10 loci)	Number of MHC alleles	Number of funct. MHC alleles	Prop. of funct. MHC alleles shared in parents (ASIN)	Distance of MHC alleles in parents (ASIN)	Distance of funct. MHC alleles in parents (ASIN)	FA00217 *	FA00275 *	FA09776 *	FA18621 *	FA21013 *	FA23304 *	FA25259 *	FA25614 *	FA25663*	FA25835 *
1	1	1	1	3	15.50	14.90	25.10	17.72	3	100.00	4	4	0.25	0.39	0.57	1	0	0	0	0	0	0	0	0	0
2	1	1	1	3	16.10	14.02	25.90	17.92	3	100.00	5	5	0.25	0.39	0.57	1	0	1	0	0	0	0	0	0	0
3	1	1	1	3	13.90	13.30	24.70	18.69	3	90.00	3	3	0.25	0.39	0.57	0	0	0	0	0	1	1	0	0	0
4	2	1	3	6	9.70	11.58	19	17.5	3	95.00	4	4	0.25	0.39	0.57	1	0	0	0	0	0	0	0	0	0
5	1	1	4	6	8.20	10.30			2	100.00	3	3	0.25	0.39	0.57	0	0	0	0	0	1	1	0	0	0
6	1	1	4	6	5.90	8.30			2	95.00	3	3	0.25	0.39	0.57	0	0	0	0	0	1	1	0	0	0
7	1	1	4	6	11.20	11.86			2	95.00	5	5	0.25	0.39	0.57	1	0	1	0	0	1	1	0	0	0
8	1	1	2	5					0	94.44	6	6	0.25	0.39	0.57	1	0	1	0	0	1	1	0	0	0
9	1	1	2	5					1	95.00	6	6	0.25	0.39	0.57	1	0	1	0	0	1	1	0	0	0
10	2	1	2	5					1	95.00	6	6	0.25	0.39	0.57	1	0	1	0	0	1	1	0	0	0
11	1	2	1	5	11.60	12.63	24.30	18.39	3	95.00	7	5	0.20	0.36	0.73	1	1	0	1	0	0	1	0	0	0
12	2	2	1	5	10.10	10.91	21.90	17.37	3	95.00	4	4	0.20	0.36	0.73	0	1	0	1	0	0	0	0	0	0
13	2	2	1	5	13.90	14.40	20.20	18.02	3	100.00	6	6	0.20	0.36	0.73	1	0	0	0	1	0	0	0	1	0
14	1	2	3	5	13.60	13.67	22.90	19.20	3	95.00	6	6	0.20	0.36	0.73	1	0	0	0	1	0	0	0	1	0
15	2	2	3	5	7.70	10.40	18.50	16.38	3	90.00	8	6	0.20	0.36	0.73	1	0	0	0	1	0	1	0	1	0
16	1	2	1	5	12.50	13.37	21.8	17.88	3	95.00	3	3	0.20	0.36	0.73	0	1	0	1	0	0	0	0	0	0
17	1	2	3	5	6.70	9.50			2	90.00	8	6	0.20	0.36	0.73	1	0	0	0	1	0	1	0	1	0
18	1	2	2	6					1	94.44	7	5	0.20	0.36	0.73	1	1	0	1	0	0	1	0	0	0
19	1	2	3	5					1	100.00	8	6	0.20	0.36	0.73	1	0	0	0	1	0	1	0	1	0
20	2	3	1	3	13.30	13.32	26.10	20.23	3	80.00	7	7	0.22	0.24	0.32	0	1	0	1	1	0	0	1	1	0
21	1	3	1	3	15.50	14.80	26.70	19.39	3	90.00	6	6	0.22	0.24	0.32	0	1	0	1	1	0	0	1	1	0
22	1	3	1	3	15.40	13.38	25.8	19.36	3	100.00	6	6	0.08	0.24	0.32	1	0	0	0	1	1	0	1	1	0
23	2	3	3	6	9.00	12.81	17.4	16.07	3	95.00	5	5	0.22	0.24	0.32	1	0	0	0	1	0	0	1	1	0
24	2	3	3	6	7.00	10.70	10.8	14.2	3	85.00	4	4	0.22	0.24	0.32	0	1	0	1	1	0	0	0	0	0
25	1	3	3	6					1	80.00	5	5	0.22	0.24	0.32	0	1	0	1	1	0	0	0	0	0

26	1	3	3	6					1	85.00	5	5	0.22	0.24	0.32	1	0	0	0	1	0	0	1	1	0
27	1	3	3	6					1	88.89	4	4	0.22	0.24	0.32	1	0	0	0	1	0	0	1	0	0
28	1	3	3	6					0	100.00	4	4	0.22	0.24	0.32	1	0	0	0	1	0	0	1	0	0
29	1	4	1	4	17.30	14.04	24.40	18.37	3	83.33	2	2	0.52	0.23	0.59	0	0	1	0	0	0	0	0	0	1
30	2	4	1	4	16.40	16.40	24.10	18.40	3	80.00	2	2	0.52	0.23	0.59	1	0	0	0	0	0	0	0	0	1
31	1	4	3	5	9.60	11.50	17.60	17.27	3	94.44	3	3	0.52	0.23	0.59	1	0	0	0	0	0	0	0	0	1
32	1	4	1	4	18.00	15.27	24	19.28	3	77.78	2	2	0.52	0.23	0.59	1	0	0	0	0	0	0	0	0	1
33	1	4	3	5	7.80	10.50	16.4	16.4	3	75.00	2	2	0.52	0.23	0.59	0	0	1	0	0	0	0	0	0	1
34	1	4	3	5	8.90	11.60	15	16.7	3	85.00	3	3	0.52	0.23	0.59	1	0	1	0	0	0	0	0	0	0
35	2	4	3	5	5.50	9.00			2	90.00	3	3	0.52	0.23	0.59	1	0	1	0	0	0	0	0	0	1
36	2	5	1	3	16.70	14.95	24.70	19.12	3	95.00	4	4	0.25	0.19	0.42	1	0	0	0	0	1	0	0	0	0
37	1	5	1	3	14.50	13.49	24.10	19.19	3	90.00	3	3	0.25	0.19	0.42	1	0	0	0	0	0	0	1	0	0
38	1	5	1	3	17.60	14.73	29.50	19.75	3	100.00	4	4	0.25	0.19	0.42	1	0	0	0	0	0	0	0	0	0
39	1	5	3	6	19.40	15.10	27.00	18.70	3	95.00	4	4	0.11	0.19	0.42	1	0	0	0	0	1	0	0	0	0
40	1	5	3	6	16.20	13.80	24.70	18.20	3	100.00	3	3	0.25	0.19	0.42	1	0	0	0	0	0	0	1	0	0
41	1	5	3	6	19.10	14.60	27.40	18.80	3	95.00	3	3	0.11	0.19	0.42	1	0	0	0	0	1	0	1	0	0
42	2	5	3	6	11.50	13.19	20.60	16.70	3	95.00	3	3	0.25	0.19	0.42	1	0	0	0	0	0	0	1	0	0
43	1	5	2	6	7.00	10.60	11.10	13.91	3	100.00	4	4	0.25	0.19	0.42	1	0	0	0	0	1	0	1	0	0
44	1	5	3	6	8.10	9.75			2	85.00	5	5	0.11	0.19	0.42	1	0	0	0	1	0	1	1	0	0
45	1	6	1	3	19.60	16.51	24.60	19.60	3	85.00	3	3	0.22	0.39	0.73	1	0	0	0	0	0	0	0	0	1
46	1	6	1	3	14.70	14.55	21.60	17.67	3	100.00	2	2	0.22	0.39	0.73	0	0	0	0	0	0	0	0	1	0
47	1	6	1	3	15.10	14.40	22.30	18.23	3	100.00	4	4	0.22	0.39	0.73	0	0	0	0	1	0	0	1	1	0
48	1	6	3	5	11.20	12.40	19.30	17.95	3	90.00	4	4	0.22	0.39	0.73	1	0	0	0	0	0	0	0	0	1
49	1	6	3	5	12.80	13.55	22	18.3	3	85.00	6	6	0.22	0.39	0.73	1	0	0	0	1	0	0	1	0	1
50	1	6	3	5	9.80	12.04			2	95.00	2	2	0.22	0.39	0.73	0	0	0	0	0	0	0	0	1	0
51	1	6	2	5					E	90.00	4	4	0.22	0.39	0.73	0	0	0	0	1	0	0	1	1	0
52	2	7	1	3	9.20	12.22	16.60	16.39	3	95.00	4	4	0.29	0.27	0.44	1	0	1	0	0	0	0	0	1	0
53	1	7	1	3	8.30	11.89	17.20	16.17	3	95.00	6	5	0.29	0.27	0.44	1	1	1	0	0	0	0	0	0	0
54	1	7	1	3	9.90	11.95	19.00	18.07	3	100.00	6	5	0.29	0.27	0.44	1	1	1	0	0	0	0	0	0	0
55	1	7	2	5	12.00	13.30	23.8	18.33	3	95.00	6	5	0.29	0.27	0.44	1	1	1	0	0	0	0	0	0	0
56	1	7	2	5					1	100.00	7	6	0.29	0.27	0.44	1	0	1	0	0	0	0	0	1	0
57	1	8	1	4	12.20	12.70	24.90	17.99	3	95.00	6	6	0.22	0.31	0.48	0	0	1	0	1	0	0	0	1	0
58	1	8	1	4	13.50	13.59	26.10	18.90	3	95.00	3	3	0.22	0.31	0.48	0	0	0	1	1	1	0	0	0	0
59	1	8	3	5	15.50	13.40	27.2	18.42	3	85.00	6	6	0.22	0.31	0.48	0	0	1	0	1	0	0	0	1	0
60	2	8	3	5					0	100.00	5	5	0.22	0.31	0.48	0	0	0	1	0	1	0	0	1	0
61	1	8	2	6					0	90.00	3	3	0.22	0.31	0.48	0	0	1	0	1	0	0	0	0	0
62	1	8	3	5					0	90.00	3	3	0.22	0.31	0.48	0	0	1	0	1	0	0	0	0	0

63	2	9	1	5	9.20	11.80	21.80	17.50	3	80.00	4	4	0.20	0.33	0.42	1	0	0	0	1	0	0	1	0	0
64	2	9	1	5	9.20	11.38	18.70	18.39	3	94.44	7	7	0.20	0.33	0.42	1	1	0	1	1	0	1	0	0	0
65	1	9	1	5	11.00	12.25	23.00	17.75	3	100.00	5	5	0.20	0.33	0.42	0	1	0	1	1	0	0	0	0	0
66	1	9	1	5	5.10	9.55	13.50	15.10	3	90.00	4	4	0.20	0.33	0.42	1	0	0	0	1	0	0	1	0	0
67	2	9	2	5					1	100.00	5	5	0.20	0.33	0.42	1	0	0	0	1	0	1	1	0	0
68	1	10	1	3	14.50	12.97	28.30	19.00	3	95.00	6	6	0.00	0.58	1.03	0	0	0	1	1	0	0	0	1	1
69	1	10	3	5	8.40	10.80	18.30	15.50	3	95.00	6	6	0.00	0.58	1.03	1	0	1	0	1	0	0	0	1	1
70	1	10	3	5	11.60	13.02	21.70	16.00	3	95.00	6	6	0.00	0.58	1.03	1	0	1	0	1	0	0	0	1	1
71	1	10	1	3	10.70	10.98	26.7	17.52	3	95.00	6	6	0.00	0.58	1.03	1	0	1	0	1	0	0	0	1	1
72	2	10	3	5	6.50	9.90			2	95.00	6	6	0.00	0.58	1.03	1	0	1	0	1	0	0	0	1	1
73	2	10	3	5					1	90.00	5	5	0.00	0.58	1.03	1	0	1	0	1	0	0	0	0	0
74	1	10	3	5					0	90.00	6	6	0.00	0.58	1.03	0	0	0	1	1	0	0	0	1	1
75	2	11	1	3	10.30	10.75	29.30	18.76	3	85.00	7	6	0.00	0.55	0.94	0	0	0	0	1	0	0	1	1	0
76	2	11	1	3	13.90	12.48	30.00	19.63	3	90.00	7	6	0.00	0.55	0.94	0	0	0	0	1	0	0	1	1	0
77	1	11	1	3	15.40	13.75	31.20	19.91	3	90.00	5	5	0.00	0.55	0.94	1	0	0	1	0	0	0	0	0	0
78	2	11	3	4	16.30	14.33	27.00	18.53	3	80.00	4	4	0.00	0.55	0.94	1	0	0	1	0	0	0	0	0	0
79	1	11	3	4	10.00	11.18	24.20	18.57	3	100.00	5	4	0.00	0.55	0.94	1	1	0	0	0	0	0	0	0	1
80	1	12	1	4	9.50	12.47	17.60	16.84	3	85.00	6	6	0.00	0.46	0.53	1	0	0	0	1	0	1	0	0	0
81	2	12	2	4	13.80	13.30	26.7	18.9	3	80.00	3	3	0.00	0.46	0.53	1	0	0	0	0	0	0	0	0	0
82	2	12	2	4	15.10	14.73	26.8	19.29	3	80.00	4	3	0.00	0.46	0.53	1	0	0	0	0	0	0	0	0	0
83	2	13	1	5	9.80	12.05	24.10	16.78	3	90.00	2	2	0.00	0.49	0.49	0	0	0	0	0	0	0	0	0	0
84	2	13	2	4	12.60	13.08	22.90	17.06	3	100.00	6	5	0.00	0.49	0.49	1	0	0	0	1	0	0	1	1	0
85	1	14	1	4	7.80	11.17	20.40	16.80	3	100.00	6	6	0.00	0.41	0.73	1	1	0	0	1	0	0	1	1	0
86	2	14	1	4	15.70	14.32	27.70	17.25	3	95.00	3	3	0.00	0.41	0.73	1	1	0	0	0	0	0	0	0	0
87	1	14	1	4	10.80	12.03	21.80	17.00	3	95.00	5	3	0.00	0.41	0.73	1	0	0	0	0	0	1	0	0	0
88	1	14	1	4	13.20	13.55	26.90	18.57	3	95.00	5	3	0.00	0.41	0.73	1	0	0	0	0	0	1	0	0	0
89	2	14	3	5	8.70	10.17	20.00	16.39	3	100.00	8	3	0.00	0.41	0.73	1	1	0	0	0	0	0	0	0	0
90	1	14	3	5	7.80	9.90	16.30	16.09	3	94.44	3	6	0.00	0.41	0.73	1	0	0	0	1	0	1	1	1	0
91	2	14	3	5	5.90	8.21	9.5	12.99	3	100.00	3	3	0.00	0.41	0.73	1	1	0	0	0	0	0	0	0	0
92	1	14	3	5	7.90	9.69	14.5	16.24	3	95.00	3	3	0.00	0.41	0.73	1	1	0	0	0	0	0	0	0	0
93	1	14	2	5					0	100.00	5	3	0.00	0.41	0.73	1	0	0	0	0	0	1	0	0	0
94	2	14	3	5					0	100.00	7	4	0.00	0.41	0.73	1	1	0	0	0	0	1	0	0	0
95	2	14	2	5					0	94.44	8	6	0.00	0.41	0.73	1	0	0	0	1	0	1	1	1	0
96	2	15	1	3	12.40	13.24	26.10	17.77	3	95.00	6	6	0.00	0.43	0.65	1	0	1	0	1	0	1	0	1	0
97	1	15	1	3	9.40	12.46	21.90	17.23	3	85.00	4	4	0.00	0.43	0.65	1	0	0	0	1	0	1	0	0	0
98	2	15	3	3	9.50	11.00	15.4	16.3	3	90.00	4	4	0.00	0.43	0.65	1	0	1	0	0	1	0	0	1	0
99	1	15	2	3					0	88.89	6	6	0.00	0.43	0.65	1	0	1	0	0	1	0	0	1	0

100	2	15	2	3					0	90.00	4	4	0.00	0.43	0.65	1	0	0	0	1	0	1	0	0	0
101	2	16	1	4	11.70	13.50	23.30	17.50	3	85.00	3	3	0.20	0.49	1.06	1	0	0	0	0	0	0	0	0	0
102	1	16	1	4	11.90	13.22	22.60	18.08	3	75.00	5	5	0.20	0.49	1.06	0	0	1	0	1	0	0	0	1	0
103	1	16	1	4	10.80	13.10	22.90	18.50	3	80.00	5	5	0.20	0.49	1.06	0	0	1	0	1	0	0	0	1	0
104	1	16	1	4	15.70	14.50	23.60	19.07	3	80.00	4	4	0.20	0.49	1.06	1	0	0	0	0	1	0	0	0	0
105	1	16	2	4	11.40	13.11	19.50	17.63	3	80.00	5	5	0.20	0.49	1.06	0	0	1	0	1	0	0	0	1	0
106	2	16	2	4	10.00	11.56	19.30	16.90	3	83.33	3	3	0.20	0.49	1.06	1	0	0	0	0	0	0	0	0	0
107	1	16	2	4	14.20	13.55	23.20	18.05	3	70.00	7	7	0.20	0.49	1.06	1	0	1	0	1	1	0	0	1	0
108	1	17	1	4	9.70	11.00	22.00	18.80	3	85.00	4	4	0.29	0.37	0.73	1	1	0	0	1	0	0	0	0	0
109	2	17	1	4	11.90	11.50	21.40	17.30	3	100.00	4	4	0.29	0.37	0.73	1	1	0	0	1	0	0	0	0	0
110	2	17	1	4	8.70	9.80	18.40	17.10	3	95.00	3	3	0.29	0.37	0.73	1	0	0	0	0	0	0	0	0	0
111	2	18	1	5	13.20	12.72	23.00	18.80	3	85.00	6	6	0.25	0.47	0.91	1	0	1	0	1	0	0	0	0	0
112	1	18	1	5	7.70	9.79	21.50	17.20	3	90.00	6	6	0.25	0.47	0.91	1	0	1	0	1	0	0	0	0	0
113	2	18	1	5	10.80	10.34	22.70	18.70	3	95.00	3	2	0.25	0.47	0.91	1	0	0	0	0	0	0	0	0	0
114	1	18	1	4					0	85.00	5	5	0.25	0.47	0.91	0	0	0	0	1	0	1	0	0	0
115	1	19	1	4	11.10	11.93	21.80	17.80	3	90.00	4	4	0.00	0.34	0.32	1	0	1	0	1	0	0	0	0	0
116	1	19	1	4	10.50	11.65	23.30	18.50	3	100.00	4	4	0.00	0.34	0.32	1	0	1	0	1	0	0	0	0	0
117	1	19	1	4	12.90	12.76	23.00	18.62	3	90.00	4	3	0.00	0.34	0.32	1	0	1	0	0	0	0	0	0	0
118	1	19	2	5	11.20	12.98	22.90	18.30	3	80.00	4	3	0.00	0.34	0.32	1	0	0	1	0	0	0	0	1	0
119	1	19	3	4	14.90	11.75	28.10	18.61	3	95.00	3	2	0.00	0.34	0.32	1	0	1	0	0	0	0	0	0	0
120	1	19	1	4	8.00	10.10	19.40	17.25	3	90.00	3	2	0.00	0.34	0.32	1	0	1	0	0	0	0	0	0	0
121	1	19	2	5	7.10	10.31	12.5	15	3	90.00	5	5	0.00	0.34	0.32	1	0	0	1	1	0	0	0	1	0
122	1	19	2	5					0	94.44	5	5	0.00	0.34	0.32	1	0	0	1	1	0	0	0	1	0
123	2	20	1	4	12.10	11.81	22.70	17.90	3	95.00	5	3	0.41	0.43	0.84	1	1	1	0	0	0	0	0	0	0
124	1	20	1	4	7.30	10.90	17.5	17.18	3	95.00	3	3	0.41	0.43	0.84	1	1	1	0	0	0	0	0	0	0
125	2	20	2	5	8.20	11.40			2	90.00	3	3	0.41	0.43	0.84	1	1	1	0	0	0	0	0	0	0
126	2	21	1	5	16.70	15.09	27.60	18.60	3	100.00	5	4	0.25	0.29	0.48	1	0	0	0	0	1	0	0	0	0
127	2	21	1	5	10.40	12.13	23.60	18.01	3	88.89	5	4	0.25	0.29	0.48	1	0	0	0	0	0	0	1	0	0
128	2	21	1	5	16.70	14.94	27.40	18.53	3	94.44	5	4	0.25	0.29	0.48	1	0	0	0	0	1	0	0	0	0
129	2	21	1	5	14.40	14.88	27.9	18.3	3	94.44	5	4	0.25	0.29	0.48	1	0	0	0	0	1	0	0	0	0
130	1	22	1	5	12.40	13.63	24.30	18.58	3	100.00	5	4	0.52	0.21	0.71	1	0	0	0	1	0	0	0	0	1
131	1	22	2	6	12.40	12.91	23.20	17.31	3	85.00	3	2	0.52	0.21	0.71	1	0	0	0	0	0	0	1	0	0
132	2	22	1	5	10.60	12.65	19.7	17.41	3	88.89	5	3	0.52	0.21	0.71	1	0	0	0	0	0	0	0	0	1
133	2	22	1	5	10.60	12.65	19.7	17.41	3	100.00	3	2	0.52	0.21	0.71	1	0	0	0	0	0	0	1	0	0
134	2	22	1	5	9.90	11.37	21.6	18.8	3	88.89	5	3	0.52	0.21	0.71	1	0	0	0	0	0	0	0	0	1
135	1	22	2	6	8.90	10.80			2	95.00	3	3	0.52	0.21	0.71	0	0	0	0	1	0	0	1	0	1
136	2	22	2	6	12.70	13.57			2	90.00	3	2	0.52	0.21	0.71	1	0	0	0	0	0	0	1	0	0

137	1	22	2	6					1	90.00	3	2	0.52	0.21	0.71	1	0	0	0	0	0	0	1	0	0
138	2	23	2	5	11.20	13.20	20.30	18.66	3	90.00	4	4	0.46	0.42	0.64	1	0	1	0	0	1	0	0	1	0
139	1	23	1	5	11.50	11.29	24.00	19.38	3	85.00	4	4	0.46	0.42	0.64	1	0	1	0	0	1	0	0	1	0
140	2	23	1	5	15.40	15.17	21.90	19.99	3	90.00	5	5	0.46	0.42	0.64	1	0	1	0	1	0	1	0	0	0
141	2	23	1	5	16.60	14.73	24.90	19.69	3	85.00	6	6	0.46	0.42	0.64	1	0	1	0	1	0	1	0	0	0
142	2	23	1	5	15.40	14.14	23.20	18.60	3	95.00	5	5	0.46	0.42	0.64	1	0	1	0	0	1	0	0	1	0
143	2	24	1	4	11.30	11.90	22.00	17.14	3	94.44	5	5	0.00	0.54	0.45	1	1	1	0	0	1	0	0	0	0
144	1	24	2	5	8.50	10.50	22.70	17.70	3	90.00	5	5	0.00	0.54	0.45	1	1	1	0	0	1	0	0	0	0
145	2	24	2	5	7.20	9.83	18.50	16.70	3	90.00	5	5	0.00	0.54	0.45	0	0	0	0	1	0	1	0	0	0
146	2	24	1	4	8.80	10.10	18.1	15.8	3	83.33	5	5	0.00	0.54	0.45	1	1	1	0	0	1	0	0	0	0
147	1	24	2	5					1	95.00	8	8	0.00	0.54	0.45	0	1	1	0	1	0	1	0	0	0
148	1	24	2	5					0	100.00	5	5	0.00	0.54	0.45	1	1	1	0	0	1	0	0	0	0
149	1	25	1	3	9.40	10.90	20.20	17.27	3	88.89	5	4	0.25	0.43	0.92	1	0	0	0	0	0	0	0	0	1
150	2	25	1	3	8.30	10.58	12.00	14.35	3	94.44	5	4	0.17	0.43	0.92	1	0	1	0	1	0	0	1	0	0
151	1	25	1	3	7.60	11.07	12	15.05	3	100.00	7	6	0.25	0.43	0.92	1	0	1	0	1	0	0	1	0	0
152	1	26	1	4	11.60	10.77	24.20	16.89	3	100.00	5	4	0.20	0.50	0.67	0	0	0	0	1	0	0	1	0	0
153	1	27	1	5	14.20	13.80	25.70	18.50	3	95.00	7	6	0.22	0.48	0.88	1	0	1	0	0	0	0	0	0	0
154	2	27	1	5	7.60	11.32	21.20	16.39	3	90.00	5	5	0.22	0.48	0.88	1	0	1	0	0	1	0	0	0	0
155	1	27	2	4	14.10	12.60	24.40	17.03	3	90.00	3	3	0.22	0.48	0.88	1	0	0	0	0	1	0	0	0	0
156	2	27	1	5	7.60	11.32			2	85.00	5	5	0.22	0.48	0.88	1	0	1	0	0	1	0	0	0	0
157	1	27	2	4					1	95.00	4	4	0.22	0.48	0.88	1	0	0	0	0	1	0	0	0	0
158	1	27	2	4					0	100.00	5	5	0.22	0.48	0.88	1	0	1	0	0	1	0	0	0	0
159	1	27	2	4					1	90.00	4	4	0.22	0.48	0.88	1	0	0	0	0	1	0	0	0	0
160	1	28	1	3	8.00	10.40	18.50	15.99	3	80.00	6	5	0.22	0.45	0.85	1	0	0	0	1	0	0	0	0	0
161	1	28	1	3	9.90	11.42	24.3	18.04	3	100.00	3	3	0.11	0.45	0.85	1	1	0	0	0	0	0	0	0	0
162	2	28	1	3					0	90.00	4	4	0.22	0.45	0.85	1	1	0	0	1	0	0	0	0	0
163	2	28	2	3					0	90.00	4	4	0.22	0.45	0.85	1	1	0	0	1	0	0	0	0	0
164	2	28	2	3					0	95.00	4	4	0.22	0.45	0.85	1	1	0	0	1	0	0	0	0	0
165	1	29	3	5	12.40	12.80	21.20	17.20	3	100.00	3	2	0.29	0.36	0.19	1	0	0	0	0	0	0	0	0	0
166	1	29	3	5	14.90	13.07	29.90	18.50	3	100.00	4	3	0.29	0.36	0.19	1	1	0	0	0	0	0	0	0	0
167	1	29	1	4	18.20	14.49	29.50	19.73	3	100.00	2	2	0.29	0.36	0.19	0	1	0	0	0	0	0	0	0	0
168	2	29	1	4	16.00	13.13	28.30	19.55	3	95.00	2	2	0.29	0.36	0.19	0	1	0	0	0	0	0	0	0	0
169	1	29	2	6					1	95.00	4	4	0.29	0.36	0.19	1	1	0	0	0	0	0	0	1	0
170	1	29	2	6					0	95.00	3	2	0.29	0.36	0.19	1	0	0	0	0	0	0	0	0	0
171	2	30	3	6	9.10	11.20	18.40	16.60	3	80.00	5	3	0.29	0.40	0.75	1	0	1	0	0	0	0	0	0	0
172	1	30	3	6	9.10	11.95	20.50	17.58	3	90.00	5	3	0.29	0.40	0.75	1	0	1	0	0	0	0	0	0	0
173	2	30	3	6	10.20	11.09	19.50	17.22	3	85.00	5	3	0.29	0.40	0.75	1	0	1	0	0	0	0	0	0	0

174	1	30	3	6	8.50	10.60	17.60	16.40	3	90.00	5	3	0.29	0.40	0.75	1	0	1	0	0	0	0	0	0	0
175	1	30	1	4	8.20	10.55			2	85.00	4	4	0.29	0.40	0.75	1	0	0	0	0	1	0	0	0	0
176	1	30	1	4	7.10	8.51			2	90.00	5	4	0.29	0.40	0.75	1	0	1	0	0	0	0	0	0	0
177	1	30	1	4	11.90	11.29	19.7	16.6	3	80.00	4	4	0.29	0.40	0.75	1	0	0	0	0	1	0	0	0	0
178	2	30	3	6	4.10	8.50	5.5	11.9	3	75.00	4	4	0.29	0.40	0.75	1	0	0	0	0	1	0	0	0	0
179	2	30	2	5	4.30	7.10			2	95.00	5	3	0.29	0.40	0.75	1	0	1	0	0	0	0	0	0	0
180	2	31	1	3	13.70	13.70	19.70	17.04	3	80.00	6	5	0.46	0.27	0.53	1	0	0	1	0	0	0	0	0	0
181	1	31	1	3	13.90	11.46	20.70	17.75	3	88.89	3	3	0.46	0.27	0.53	1	0	1	1	0	0	0	0	0	0
182	1	31	1	2					1	95.00	3	3	0.46	0.27	0.53	1	0	1	0	0	0	0	0	0	0
183	2	32	1	4	11.30	11.80	22.10	16.52	3	95.00	3	2	0.29	0.45	0.62	1	0	0	0	0	0	0	0	0	0
184	2	32	1	4	13.20	13.10	23.50	17.24	3	95.00	3	2	0.29	0.45	0.62	1	0	0	0	0	0	0	0	0	0
185	1	32	1	4	13.80	13.00	23.90	18.12	3	100.00	3	2	0.29	0.45	0.62	1	0	0	0	0	0	0	0	0	0
186	1	33	1	4	8.30	10.30	21.20	15.77	3	95.00	5	4	0.29	0.45	0.62	1	1	0	0	0	0	0	0	0	1
187	1	33	1	4	7.60	9.90	16.30	15.71	3	88.89	5	4	0.29	0.45	0.62	1	1	0	0	0	0	0	0	0	1
188	2	33	1	4	10.10	11.60	21.40	16.62	3	90.00	4	3	0.29	0.45	0.62	1	1	1	0	0	0	0	0	0	0
189	1	34	2	5	9.10	9.60	17.10	15.92	3	95.00	3	2	0.29	0.45	0.97	1	0	1	0	0	0	0	0	0	0
190	1	34	2	5	13.90	11.90	27.40	18.40	3	95.00	6	4	0.29	0.45	0.97	1	0	0	0	0	0	0	0	0	0
191	1	34	2	5					1	100.00	5	5	0.29	0.45	0.97	1	0	0	0	0	0	0	0	0	0
192	2	34	2	5	10.80	11.50			2	100.00	3	3	0.29	0.45	0.97	1	0	1	0	0	0	0	0	0	0
193	2	34	2	5					0	95.00	5	4	0.29	0.45	0.97	1	0	1	0	0	0	0	0	0	0
194	2	35	3	4	10.40	12.40	22.90	16.34	3	85.00	6	6	0.20	0.41	0.97	1	0	0	1	1	0	0	1	0	0
195	1	35	1	5	6.40	9.31			2	90.00	5	5	0.20	0.41	0.97	0	0	0	1	1	0	0	0	0	0
196	1	35	3	4					1	95.00	5	5	0.20	0.41	0.97	0	0	0	1	1	0	0	0	0	0
197	1	35	3	4					1	95.00	5	5	0.20	0.41	0.97	1	0	1	0	1	0	0	0	0	0
198	1	35	3	4					0	80.00	5	5	0.20	0.41	0.97	1	0	1	0	1	0	0	0	0	0
199	2	36	3	5	10.00	11.32	19.00	15.42	3	90.00	1	1	0.41	0.29	0.77	0	0	0	0	0	0	0	0	0	0
200	1	36	3	5	9.90	11.29	15.90	15.45	3	100.00	3	3	0.41	0.29	0.77	1	0	0	0	0	0	0	0	0	0
201	1	36	3	5					1	90.00	3	3	0.41	0.29	0.77	1	0	0	0	0	0	0	0	0	0
202	1	36	3	5	7.80	10.46	11.6	14.68	3	95.00	5	3	0.41	0.29	0.77	1	0	0	0	0	0	0	0	0	0
203	2	36	3	5					1	95.00	5	3	0.41	0.29	0.77	1	0	0	0	0	0	0	0	0	0
204	1	37	2	4	10.00	10.70	21.40	16.90	3	90.00	5	4	0.52	0.28	0.48	1	1	0	0	0	1	0	0	1	0
205	2	37	2	4	8.60	10.90	17.00	16.00	3	90.00	7	7	0.52	0.28	0.48	1	1	1	0	1	0	1	0	0	0
206	1	37	2	4	10.60	10.54	20.60	16.15	3	90.00	3	3	0.34	0.28	0.48	1	1	0	0	0	0	0	0	0	1
207	1	38	2	4	11.60	13.04	20.40	16.30	3	95.00	5	4	0.34	0.38	0.87	1	0	0	0	1	0	0	0	0	0
208	2	38	2	4	11.30	12.21	19.40	17.40	3	95.00	3	3	0.34	0.38	0.87	0	0	0	0	1	0	0	0	0	0
209	1	38	2	4	9.10	11.15	18.00	16.46	3	95.00	3	2	0.34	0.38	0.87	1	0	0	0	0	0	1	0	0	0
210	2	39	4	6	10.80	12.28	19.80	17.40	3	80.00	6	6	0.34	0.38	0.31	1	1	0	1	1	0	0	0	0	0

211	1	39	4	6	13.00	12.77	22.70	17.30	3	85.00	6	6	0.34	0.38	0.31	1	1	0	1	1	0	0	0	0	0
212	1	39	4	6	11.80	11.86	19.6	17.2	3	80.00	6	6	0.34	0.38	0.31	1	1	0	1	1	1	0	0	0	0
213	2	39	4	6					0	80.00	5	5	0.34	0.38	0.31	0	0	1	0	1	0	1	1	0	0
214	2	39	4	6					0	90.00	6	6	0.34	0.38	0.31	1	1	0	1	1	1	0	0	0	0
215	2	40	1	4	16.80	13.41	24.90	17.93	3	85.00	4	4	0.25	0.31	0.43	1	0	0	0	0	0	0	0	1	0
216	1	40	1	4	16.40	13.41	27.00	18.72	3	85.00	4	4	0.25	0.31	0.43	1	0	0	0	0	0	0	0	1	0
217	1	40	3	5	13.10	12.48	27.3	18.68	3	85.00	5	5	0.25	0.31	0.43	1	0	0	0	0	0	0	0	1	0
218	2	40	3	5	10.30	10.73	25	17.7	3	90.00	5	5	0.25	0.31	0.43	1	0	0	0	0	0	0	0	1	0
219	2	40	2	4					0	85.00	5	5	0.25	0.31	0.43	1	0	0	0	0	0	0	0	1	0
220	1	41	1	6	14.70	13.68	27.00	17.73	3	90.00	6	6	0.30	0.34	0.29	1	0	1	1	1	1	0	0	0	0
221	2	41	1	6	14.80	13.68	27.50	18.53	3	95.00	6	6	0.30	0.34	0.29	1	0	1	1	1	1	0	0	0	0
222	2	41	1	6	13.40	13.28	27.40	17.33	3	100.00	6	6	0.30	0.34	0.29	1	0	1	1	1	1	0	0	0	0
223	1	41	1	6	11.20	12.02	28.8	17.56	3	85.00	6	6	0.48	0.34	0.29	1	1	0	1	1	0	0	0	0	0
224	2	41	2	7	9.90	11.70	20.7	17.2	3	95.00	7	7	0.48	0.34	0.29	1	1	1	1	1	1	0	0	0	0
225	1	41	1	4					1	90.00	4	4	0.48	0.34	0.29	1	0	0	0	0	1	1	1	0	0
226	2	41	1	6					1	94.44	7	7	0.48	0.34	0.29	1	1	1	1	1	1	0	0	0	0
227	2	42	1	4	13.90	12.42	26.20	18.04	3	90.00	4	4	0.00	0.32	0.66	1	0	0	0	0	1	0	0	0	0
228	2	42	1	4	11.00	10.44	21.4	17.16	3	85.00	3	3	0.00	0.32	0.66	1	0	0	0	0	1	0	0	0	0
229	1	42	3	5					1	75.00	3	3	0.00	0.32	0.66	1	0	1	0	0	0	0	0	0	0
230	2	42	3	5	6.30	9.30	13.7	14.33	3	95.00	4	4	0.00	0.32	0.66	1	0	0	0	0	1	0	0	0	0
231	2	42	3	5	7.50	10.40			2	95.00	6	6	0.00	0.32	0.66	1	0	0	0	1	0	0	1	0	1
232	2	42	3	5	7.50	10.30	16.1	15.33	3	90.00	3	3	0.00	0.32	0.66	1	0	0	0	0	1	0	0	0	0
233	1	43	1	4	10.50	12.13	21.7	16.4	3	85.00	5	4	0.00	0.47	0.83	1	0	0	0	0	0	0	0	0	0
234	2	43	1	4	7.70	10.95	16.9	15.88	3	100.00	3	3	0.00	0.47	0.83	0	0	0	0	0	0	0	0	0	0
235	2	43	1	4	7.10	11.20	14.8	15.25	3	95.00	5	4	0.00	0.47	0.83	1	0	0	0	0	0	0	0	0	0
236	2	43	1	4	9.90	12.16	16.9	15.8	3	90.00	5	4	0.00	0.47	0.83	1	0	0	0	0	0	0	0	0	0
237	1	43	3	4	8.10	10.09	17.3	15	3	85.00	5	4	0.00	0.47	0.83	1	0	0	0	0	0	0	0	0	0
238	2	43	3	4	7.80	10.00			2	95.00	4	3	0.00	0.47	0.83	1	0	0	0	0	0	0	0	0	1
239	1	43	3	4	7.30	9.40			2	90.00	5	4	0.00	0.47	0.83	1	0	0	0	0	0	0	0	0	0
240	1	43	3	4	5.40	9.40			2	95.00	5	4	0.00	0.47	0.83	1	0	0	0	0	0	0	0	0	1
241	2	44	1	4	9.40	12.33	16.9	16.21	3	100.00	6	5	0.29	0.27	0.70	1	0	0	0	0	0	0	0	0	0
242	1	44	2	5					1	95.00	4	3	0.29	0.27	0.70	1	0	0	0	0	0	0	0	0	0
243	1	44	1	4	10.20	11.81	20.2	16.9	3	95.00	3	3	0.20	0.27	0.70	1	0	0	0	0	0	0	0	0	0
244	1	44	1	4	12.60	13.13	19.2	16.4	3	90.00	2	2	0.29	0.27	0.70	1	0	0	0	0	0	0	0	0	0
245	1	44	2	5					1	90.00	4	3	0.29	0.27	0.70	1	0	0	0	0	0	0	0	0	0
246	1	44	2	5					1	95.00	5	4	0.29	0.27	0.70	1	0	0	0	0	0	0	0	0	0
247	1	44	2	5					1	90.00	5	4	0.29	0.27	0.70	1	0	0	0	0	0	0	0	0	0

248	1	44	1	4	8.40	10.72			2	90.00	5	4	0.29	0.27	0.70	1	0	0	0	0	0	0	0	0	0
249	2	45	1	4	10.30	11.02	23.4	18.08	3	95.00	5	5	0.37	0.26	0.41	1	0	0	1	0	0	0	0	0	0
250	1	45	1	4	6.00	7.40	19.1	14.34	3	80.00	6	4	0.37	0.26	0.41	1	0	1	0	0	0	0	0	0	0
251	1	45	1	4	9.40	11.25	23.7	16.7	3	85.00	7	5	0.37	0.26	0.41	1	0	1	0	0	0	0	0	0	0
252	1	45	2	3					0	90.00	7	7	0.37	0.26	0.41	1	0	1	1	1	0	0	1	0	0
253	1	45	2	3					0	83.33	6	5	0.37	0.26	0.41	1	0	0	1	0	0	0	0	0	0
254	1	46	1	5					1	100.00	4	4	0.22	0.43	0.96	1	0	0	0	0	0	0	0	0	1
255	1	46	1	5					1	95.00	5	5	0.22	0.43	0.96	1	0	0	0	1	0	0	0	0	0
256	1	46	1	5					1	95.00	6	6	0.22	0.43	0.96	1	0	0	0	1	0	0	0	0	1
257	1	46	1	5					1	100.00	4	4	0.22	0.43	0.96	1	0	0	0	1	0	0	0	0	0
258	1	46	1	5					1	95.00	6	6	0.22	0.43	0.96	1	0	0	0	1	0	0	0	0	1
259	2	47	1	4	9.70	12.10	19	17.88	3	85.00	7	7	0.00	0.46	0.57	1	0	1	1	1	0	0	0	0	0
260	1	47	1	4	6.90	9.50	13.4	14.74	3	80.00	5	5	0.00	0.46	0.57	0	0	0	0	1	0	1	0	0	0
261	1	48	2	5	10.80	10.35	21.3	15.89	3	95.00	4	4	0.52	0.35	0.74	1	0	1	0	0	0	0	1	0	0
262	2	48	2	5	8.70	9.67	17.1	15.4	3	85.00	5	5	0.52	0.35	0.74	1	0	1	0	0	0	0	1	0	0
263	1	48	2	5	11.30	11.13	22.6	17.43	3	90.00	4	3	0.52	0.35	0.74	1	0	0	0	0	0	0	1	0	0
264	2	48	2	5	5.40	8.60	15.2	14.95	3	95.00	5	5	0.52	0.35	0.74	1	0	1	0	0	1	0	0	0	0
265	1	49	1	5	6.60	9.23			2	90.00	6	5	0.73	0.20	0.17	1	1	0	1	1	0	0	0	0	0
266	1	49	1	5					E	90.00	5	2	0.73	0.20	0.17	1	0	0	0	0	0	0	0	0	0
267	1	49	1	5					1	100.00	5	2	0.73	0.20	0.17	1	0	0	0	0	0	0	0	0	0
268	1	49	2	5					1	95.00	5	4	0.73	0.20	0.17	1	0	0	0	1	0	0	0	0	1
269	1	49	2	5					1	95.00	6	5	0.73	0.20	0.17	1	1	0	1	1	0	0	0	0	0
270	1	49	2	5					0	95.00	5	4	0.73	0.20	0.17	1	0	0	0	1	0	0	0	0	1
271	1	50	2	4	5.60	8.80	12.2	14.26	3	100.00	5	3	0.73	0.32	0.54	1	0	0	0	0	0	0	0	1	0
272	1	50	2	4	5.50	8.80	8.2	13.67	3	95.00	4	4	0.73	0.32	0.54	1	0	0	0	0	0	0	0	1	0
273	1	50	2	4	9.20	11.43	16	15.52	3	95.00	4	3	0.73	0.32	0.54	1	0	0	0	0	0	0	0	1	0
274	1	50	1	5					E	90.00	4	3	0.73	0.32	0.54	1	0	0	0	0	0	0	0	1	0
275	1	50	2	5					1	100.00	5	2	0.73	0.32	0.54	1	0	0	0	0	0	0	0	0	0
276	1	51	4	4	7.90	10.70	16.2	16.8	3	95.00	5	5	0.22	0.40	0.51	0	0	0	0	1	0	0	1	1	1
277	1	51	4	4	8.50	11.05	16.1	16.36	3	90.00	5	5	0.22	0.40	0.51	0	0	0	0	1	0	0	1	1	1
278	1	51	4	4	8.80	11.30	16.8	16.3	3	95.00	3	3	0.22	0.40	0.51	0	0	0	0	1	0	0	0	0	1
279	2	51	4	4					0	95.00	5	5	0.22	0.40	0.51	0	0	0	0	1	0	0	1	1	1
280	1	52	1	4					E	100.00	3	3	0.29	0.33	0.53	1	1	0	0	0	0	0	0	0	0
281	1	53	1	3					E	100.00	6	5	0.25	0.50	0.95	1	0	1	0	1	0	0	0	0	0
282	2	53	1	3					E	100.00	6	5	0.25	0.50	0.95	1	0	0	1	0	0	0	0	0	0
283	1	53	1	3					E	100.00	6	5	0.25	0.50	0.95	1	0	0	1	0	0	0	0	0	0
284	1	54	2	5					0	90.00	3	3	0.41	0.39	0.31	1	1	0	0	0	0	1	0	0	0

285	2	54	2	5					0	95.00	3	3	0.41	0.39	0.31	1	1	0	0	0	0	1	0	0	0
286	1	55	1	5					E	100.00	7	7	0.37	0.39	0.60	1	1	1	0	1	0	1	0	0	0
287	2	55	1	5					E	94.44	7	7	0.37	0.39	0.60	1	1	1	0	1	0	1	0	0	0
288	1	56	1	5					1	90.00	5	5	0.20	0.33	0.42	0	1	0	1	1	0	0	0	0	0
289	1	57	1	2					E	90.00	5	4	0.25	0.44	0.76	1	0	0	0	1	0	1	0	0	0
290	1	57	1	2					E	95.00	5	3	0.25	0.44	0.76	1	0	0	0	0	0	0	0	0	0
291	1	58	1	4	5.90	8.35			2	95.00	7	7	0.17	0.38	0.33	1	1	1	0	1	0	1	0	0	0
292	1	58	1	4	6.00	9.69			2	100.00	6	6	0.41	0.38	0.33	1	0	0	0	1	0	1	0	0	0
293	1	59	2	5					0	95.00	6	6	0.00	0.58	1.03	0	0	0	1	1	0	0	0	1	1

*only the presence/absence (1/0) data of the 10 most common functional MHC alleles were included in the analysis

' Survival status (E = excluded, 0 = died before day 1, 1 = died before day 6, 2 = died before day 12, 3 = alive after day 12)

Supplementary Table 2: Neutral genetic variation

Primer set	PCR Mix	Number of individuals	Number of alleles	Size range	Ho	He	Primer set reference
P2D/P8	1	293	2	316 & 358			Dawson et al. (2012)<br>see Griffiths et al. (1998)
Ase18 '	1	290	17	185-249	0.932	0.88	Richardson et al. (2000)<br>see Griffith et al. (2007)
Pdoμ1	1	291	18	156-200	0.81	0.849	Neumann and Wetton (1996)
Pdoμ3	1	292	18	113-181	0.905	0.883	Neumann and Wetton (1996)
Pdoμ5	1	290	18	202-264	0.844	0.847	Griffith et al. (1999)
Pdoμ6*	1						Griffith et al. (1999)
Pdo9 '	1	290	15	362-424	0.787	0.821	Griffith et al. (2007)
Pdo10	1	289	12	102-152	0.86	0.884	Griffith et al. (2007)
Pdo16A	2	291	15	270-302	0.891	0.859	Dawson et al. (2012)
Pdo17	2	290	21	192-244	0.881	0.88	Dawson et al. (2012)
Pdo19	2	291	4	173-183	0.619	0.656	Dawson et al. (2012)
Pdo22	2	292	16	92-130	0.854	0.853	Dawson et al. (2012)
Pdo27	2	292	12	224-248	0.773	0.8	Dawson et al. (2012)
Pdo40A	2	290	15	298-328	0.942	0.92	Dawson et al. (2012)

* excluded due to amplification problems
' excluded due to deviation from Hardy-Weinberg Equilibrium
Ho = observed heterozygosity
He = expected heterozygosity

Supplementary Table 3: Allele frequencies and GenBank Acc nr of the MHC class I exon 3 alleles found (range of the 65 novel alleles from this study: Pado-UA_253, 261-299 and 328-352)

Sequence identity from the program 'seqeqseq'	MHC allele names	Genbank Acc nr (NCBI BLAST)	Frequencies (%)
seq00217	Pado-UA_238	KJ825933*	58.90
seq21013	Pado-UA_239	KJ825934*	35.79
seq25566	Pado-UA_251	KJ825942*	29.45
seq25663	Pado-UA_245	KJ825936*	21.27
seq25614	Pado-UA_338	KP940330	15.75
seq25401	Pado-UA_299	KP940319	14.52
seq25986	Pado-UA_244	KJ825935*	14.31
seq00275	Pado-UA_261	KP940280	12.88
seq25835	Pado-UA_343	KP940335	12.47
seq25361	Pado-UA_246	KJ825937*	11.45
seq26078	Pado-UA_248	KJ825939*	10.43
seq25163	Pado-UA_322	JN609643.1*	9.61
seq26163	Pado-UA_257	KJ825947*	8.79
seq25202	Pado-UA_297	KP940317	8.38
seq26190	Pado-UA_267	KP940286	8.18
seq25435	Pado-UA_329	KP940321	8.18
seq07442	Pado-UA_256	KJ825946*	7.57
seq25764	Pado-UA_342	KP940334	7.36
seq08183	gb|KC585634.1|	KC585634.1*	7.16
seq25459	Pado-UA_330	KP940322	6.54
seq25907	Pado-UA_258	KJ825948*	6.54
seq25422	Pado-UA_328	KP940320	6.34
seq25483	Pado-UA_331	KP940323	6.34
seq24170	Pado-UA_294	KP940314	5.93
seq04612	Pado-UA_269	KP940288	5.93
seq25499	Pado-UA_332	KP940324	5.52
seq26010	Pado-UA_346	KP940338	5.52
seq00886	Pado-UA_262	KP940281	5.32
seq25331	Pado-UA_298	KP940318	5.32
seq26020	Pado-UA_347	KP940339	5.11
seq26092	Pado-UA_349	KP940341	5.11
seq25144	Pado-UA_295	KP940315	4.70
seq25872	Pado-UA_319	JN609642.1*	4.50
seq18381	Pado-UA_284	KP940304	4.09
seq25416	Pado-UA_254	KJ825944*	3.89
seq07562	Pado-UA_253	KP940294	3.68
seq25677	Pado-UA_339	KP940331	3.48
seq25689	Pado-UA_340	KP940332	3.48
seq13724	Pado-UA_281	KP940301	3.27
seq26130	Pado-UA_350	KP940342	3.27
seq04517	Pado-UA_268	KP940287	3.07
seq22920	Pado-UA_289	KP940309	2.86
seq06551	Pado-UA_272	KP940291	2.66

seq25531	Pado-UA_334	KP940326	2.66
seq25581	Pado-UA_250	KJ825941*	2.66
seq18342	Pado-UA_283	KP940303	2.45
seq26154	Pado-UA_352	KP940344	2.45
seq23531	Pado-UA_292	KP940312	2.25
seq12426	Pado-UA_279	KP940299	2.04
seq18845	Pado-UA_287	KP940307	2.04
seq01833	Pado-UA_266	KP940285	2.04
seq25339	Pado-UA_315	JN609647.1*	2.04
seq25159	Pado-UA_296	KP940316	1.84
seq25703	Pado-UA_247	KJ825938*	1.84
seq25751	Pado-UA_341	KP940333	1.84
seq23146	Pado-UA_252	KJ825943*	1.64
seq23304	Pado-UA_290	KP940310	1.64
seq11402	Pado-UA_277	KP940297	1.64
seq26143	Pado-UA_351	KP940343	1.64
seq13073	Pado-UA_280	KP940300	1.43
seq11982	Pado-UA_278	KP940298	1.43
seq25525	Pado-UA_333	KP940325	1.43
seq25610	Pado-UA_337	KP940329	1.23
seq18496	Pado-UA_285	KP940305	1.02
seq06555	Pado-UA_273	KP940292	1.02
seq01345	Pado-UA_264	KP940283	1.02
seq06072	Pado-UA_271	KP940290	1.02
seq01064	Pado-UA_263	KP940282	1.02
seq25843	Pado-UA_344	KP940336	1.02
seq16648	Pado-UA_255	KJ825945*	0.82
seq18621	Pado-UA_286	KP940287	0.82
seq09776	Pado-UA_276	KP940286	0.82
seq13489	Pado-UA_336	KP940328	0.82
seq04799	Pado-UA_270	KP940289	0.82
seq01198	Pado-UA_317	JN609640.1*	0.82
seq25359	Pado-UA_335	KP940327	0.82
seq23854	Pado-UA_293	KP940313	0.61
seq08911	Pado-UA_275	KP940295	0.61
seq17491	Pado-UA_282	KP940302	0.61
seq23378	Pado-UA_291	KP940311	0.61
seq07043	Pado-UA_274	KP940293	0.41
seq01384	Pado-UA_265	KP940284	0.41
seq25870	Pado-UA_345	KP940337	0.41
seq28555	Pado-UA_348	KP940340	0.41
seq21475	Pado-UA_288	KP940308	0.20

*already published genbank sequences

Supplementary Table 4: MHC alleles (nucleotides) translated in amino acid MHC alleles (AA) and functional MHC alleles (FA)

MHC alleles (85)	AA translated MHC alleles (78)	Functional MHC alleles (59)	Frequency of funct. MHC alleles (%)
Pado-UA_238, Pado-UA_266, Pado-UA_268, Pado-UA_271, Pado-UA_272, Pado-UA_274, Pado-UA_280, Pado-UA_298, Pado-UA_246, Pado-UA_254, Pado-UA_328, Pado-UA_270, Pado-UA_273, Pado-UA_253, gb|KC585634.1|, Pado-UA_299	AA00217, AA01833, AA04517, AA06072, AA06551, AA07043, AA13073, AA25331, AA25361, AA25416, AA25422	**FA00217***	**80.04**
Pado-UA_239, Pado-UA_288, Pado-UA_346	AA21013, AA21475, AA26010	**FA21013***	**40.53**
Pado-UA_276, Pado-UA_251	AA09776, AA25566	**FA09776***	**30.25**
Pado-UA_245	AA25663	**FA25663***	**21.19**
Pado-UA_261, Pado-UA_342	AA00275	**FA00275***	**19.96**
Pado-UA_286, Pado-UA_248, Pado-UA_349	AA18621, AA26078, AA26092	**FA18621***	**16.26**
Pado-UA_290, Pado-UA_244	AA23304, AA25986	**FA23304***	**15.84**
Pado-UA_269, Pado-UA_330, Pado-UA_339	AA25259, AA25459, AA25677	**FA25259***	**16.05**
Pado-UA_338	AA25614	**FA25614***	**15.64**
Pado-UA_343	AA25835	**FA25835***	**12.55**
Pado-UA_322	AA25163	FA25163	9.47
Pado-UA_257	AA26163	FA26163	8.85
Pado-UA_297	AA25202	FA25202	8.23
Pado-UA_267	AA26190	FA26190	8.23
Pado-UA_329	AA25435	FA25435	8.02
Pado-UA_256	AA07442	FA07442	7.61
Pado-UA_258	AA25907	FA25907	6.58
Pado-UA_331	AA25483	FA25483	6.38
Pado-UA_294	AA26174	FA26174	5.97
Pado-UA_332	AA25499	FA25499	5.56
Pado-UA_262	AA00886	FA00886	5.35
Pado-UA_347	AA26020	FA26020	4.94
Pado-UA_283, Pado-UA_352	AA18342, AA26154	FA18342	4.94
Pado-UA_252, Pado-UA_350	AA23146	FA23146	4.73
Pado-UA_295	AA25144	FA25144	4.73
Pado-UA_319	AA25872	FA25872	4.53
Pado-UA_284	AA18381	FA18381	4.12
Pado-UA_340	AA25689	FA25689	3.50
Pado-UA_281	AA13724	FA13724	3.29
Pado-UA_289	AA26120	FA26120	2.88
Pado-UA_334	AA25531	FA25531	2.67
Pado-UA_250	AA25581	FA25581	2.67
Pado-UA_292	AA23531	FA23531	2.26
Pado-UA_279	AA12426	FA12426	2.06
Pado-UA_315	AA25339	FA25339	2.06
Pado-UA_287	AA25979	FA25979	2.06
Pado-UA_296	AA25159	FA25159	1.85

Pado-UA_247	AA25703	FA25703	1.85
Pado-UA_341	AA25751	FA25751	1.85
Pado-UA_277	AA11402	FA11402	1.65
Pado-UA_351	AA26143	FA26143	1.65
Pado-UA_278	AA11982	FA11982	1.44
Pado-UA_333	AA25525	FA25525	1.44
Pado-UA_337	AA25610	FA25610	1.23
Pado-UA_263	AA01064	FA01064	1.03
Pado-UA_264	AA01345	FA01345	1.03
Pado-UA_285	AA18496	FA18496	1.03
Pado-UA_344	AA25843	FA25843	1.03
Pado-UA_317	AA01198	FA01198	0.82
Pado-UA_336	AA13489	FA13489	0.82
Pado-UA_255	AA16648	FA16648	0.82
Pado-UA_335	AA25359	FA25359	0.82
Pado-UA_275	AA08911	FA08911	0.62
Pado-UA_282	AA17491	FA17491	0.62
Pado-UA_291	AA23378	FA23378	0.62
Pado-UA_293	AA23854	FA23854	0.62
Pado-UA_265	AA01384	FA01384	0.41
Pado-UA_345	AA25870	FA25870	0.41
Pado-UA_348	AA28555	FA28555	0.41

*only the presence/absence (1/0) data of the 10 most common functional MHC alleles were included in the analysis

Supplementary Figure 1: Maximum-likelihood tree for all unique translated MHC sequences (amino acid sequences) (outgroup: MHC class I from chicken. GenBank Acc nr AB159063)

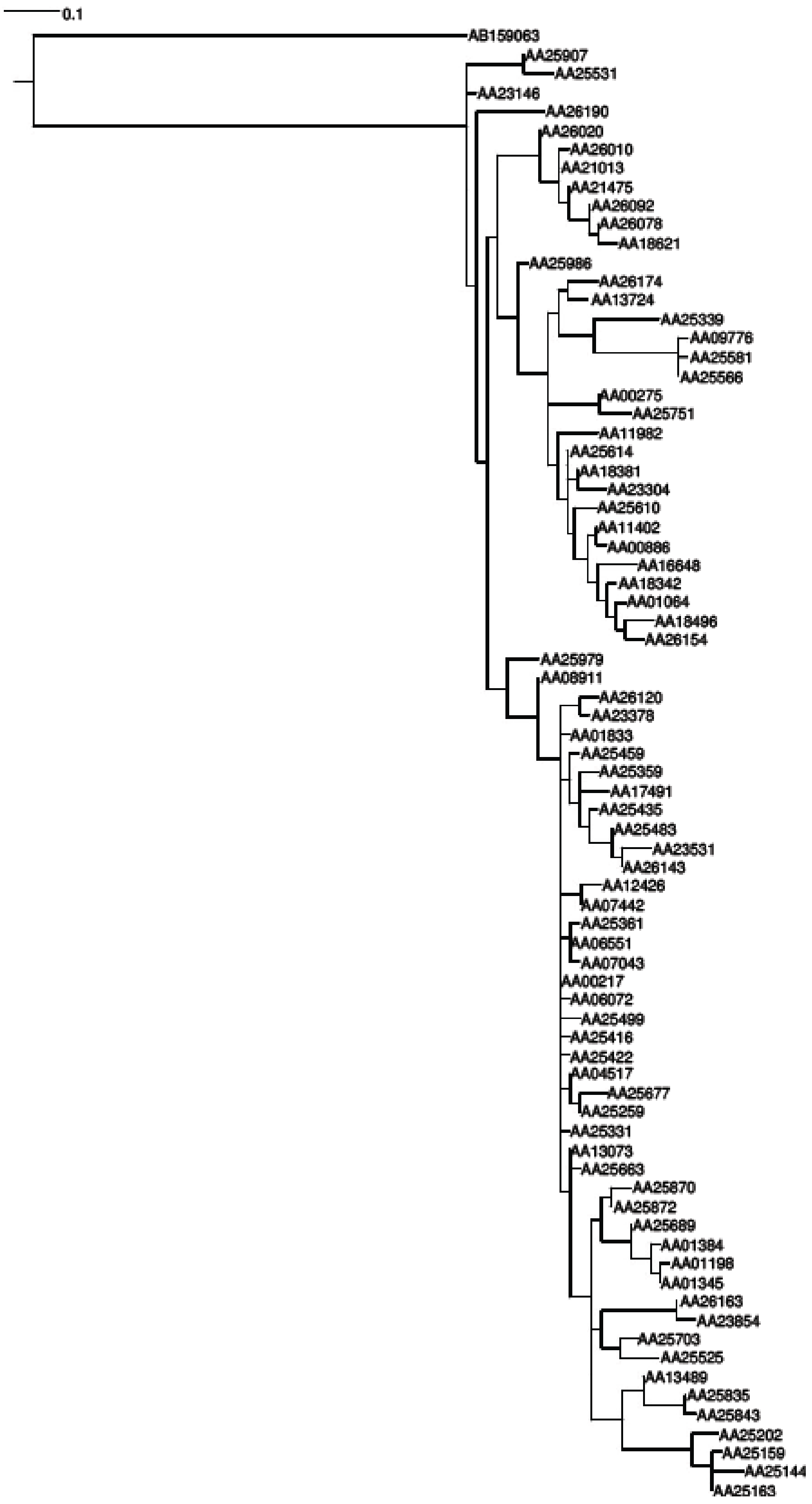

Supplementary Figure 2: Maximum-likelihood tree representing clusters of functional MHC alleles (FA) based on chemical properties in the PBR (outgroup: MHC class I from chicken. GenBank Acc nr AB159063)

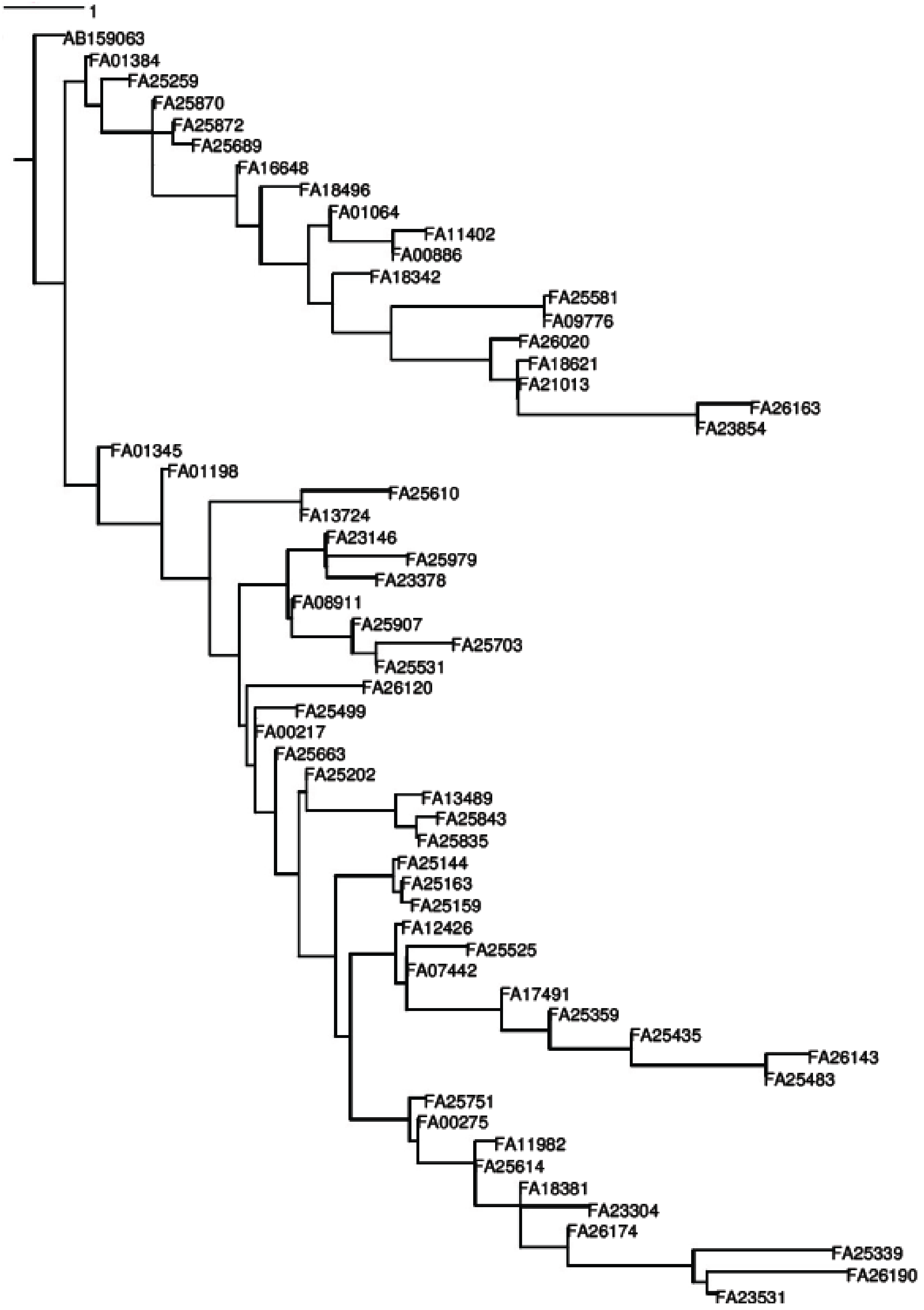

Supplementary Figure 3: Alignment of the 85 MHC alleles (nucleotides) ordered according to their frequency (most common ones on top; range of the 65 novel alleles from this study: Pado-UA_253, 261-299 and 328-352**)**

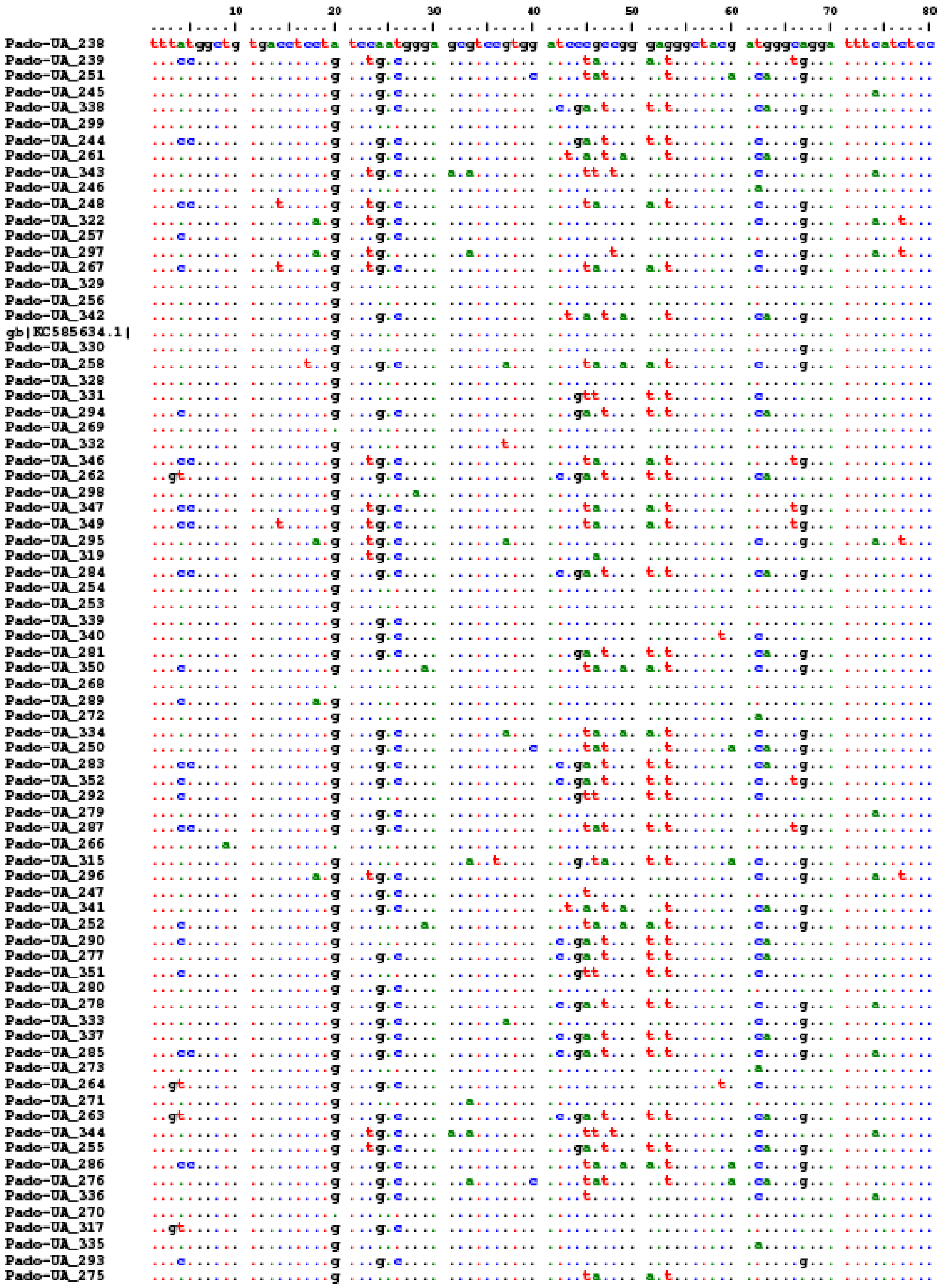

Supplementary Figure 4: Alignment of the 59 functional MHC alleles (AA) with the functional MHC allele FA25259 on top (only the first 10 functional MHC alleles that occurred in frequencies >10% were used in the analysis)

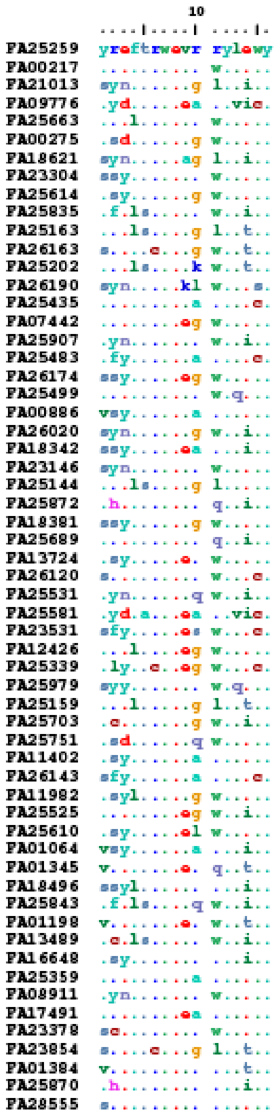

Supplementary Figure 5: Frequency distribution of the 59 functional MHC class I alleles (FA) and cut-off point for the ten most common functional alleles used in the analysis (Frequency >10%)

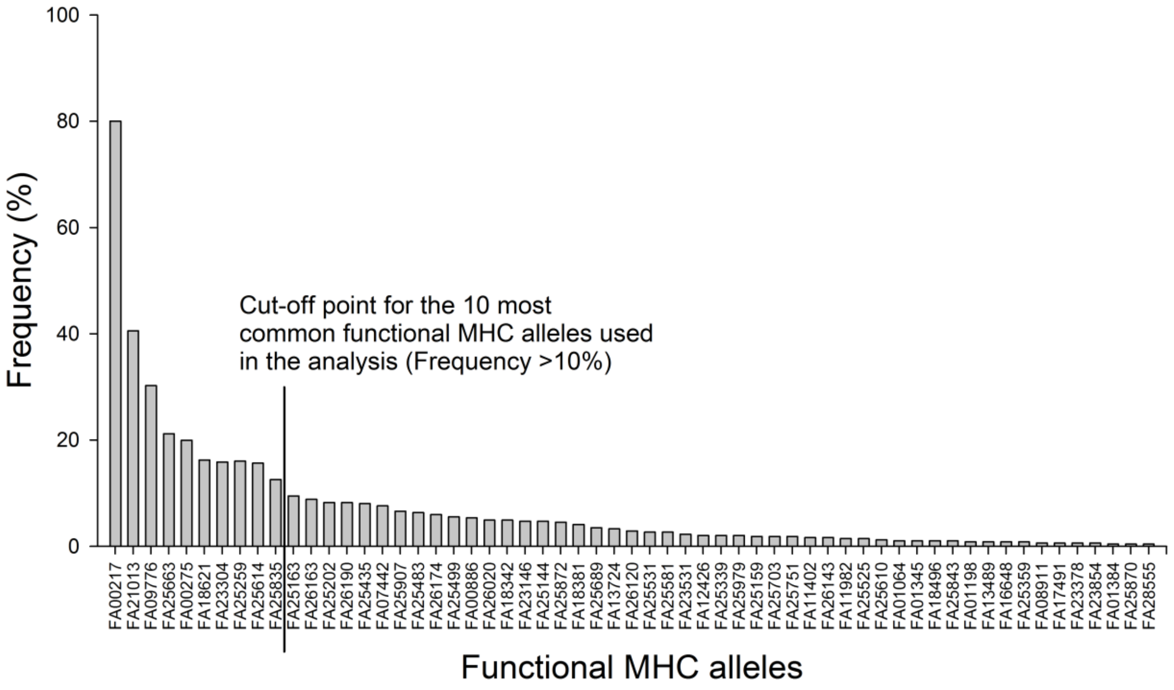

Supplementary Figure 6: Maximum-likelihood nucleotide tree representing house sparrow MHC alleles, the tree holds both new alleles from this study (*, Pado-UA_253, 261-299 and 328-352) and alleles from previous studies (retrieved from NCBI GenBank). The three alleles that code for the important FA25259 are marked (**). The present study is based on classical MHC-I alleles and the large significantly supported cluster (bt=95) consists of non-classical alleles (with a 6bp deletion) and consequently none of these alleles are represented in the present study. The tree was constructed under the GTRGAMMA model in RAxML (ver 7.0.4) with 2000 bootstraps. Bootstrap support values >60 are given.

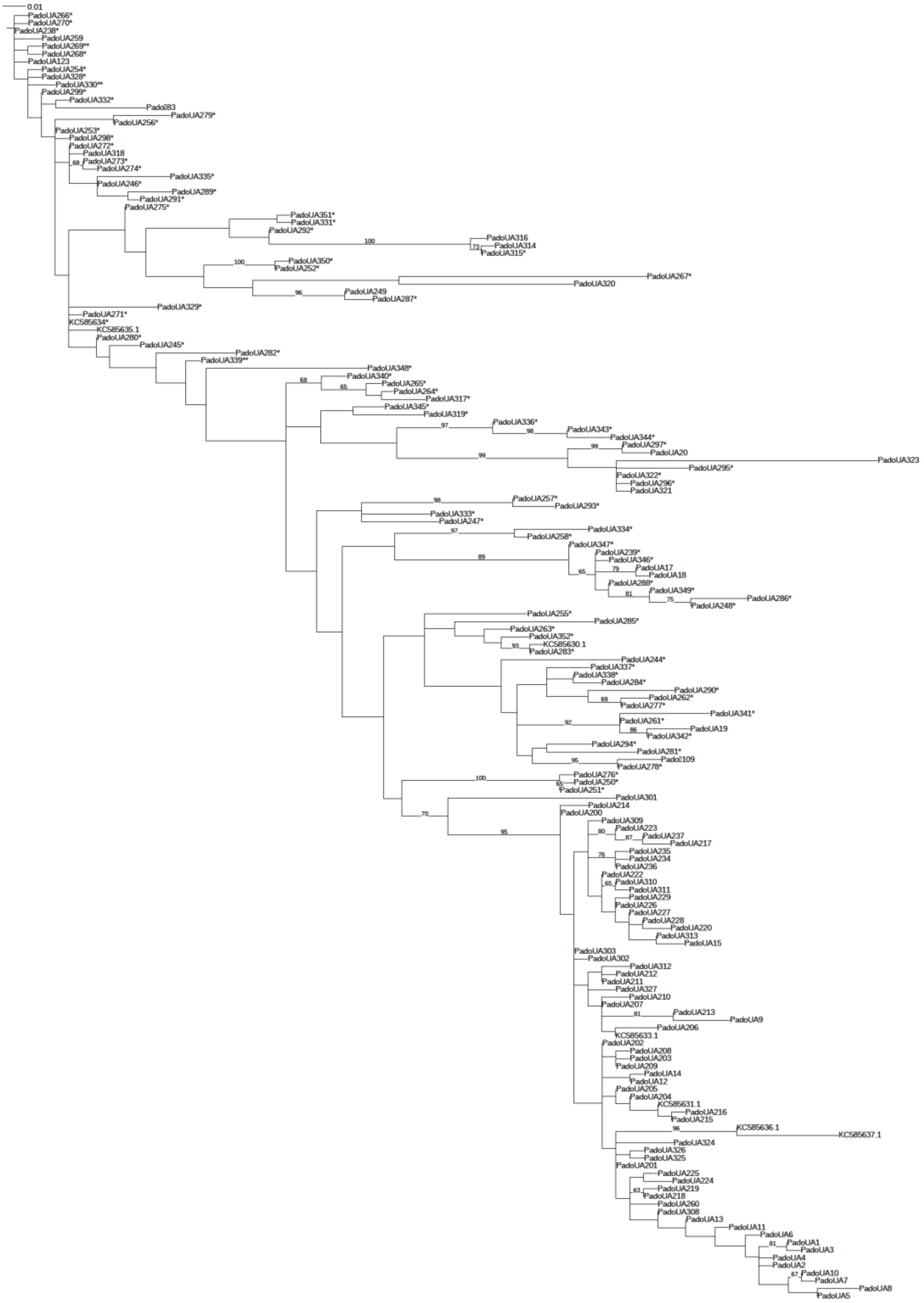

Supplementary References

Dawson DA, Horsburgh GJ, Krupa AP, *et al.* (2012) Microsatellite resources for Passeridae species: a predicted microsatellite map of the house sparrow *Passer domesticus*. *Molecular Ecology Resources*, **12**, 501-523.

Griffith SC, Dawson DA, Jensen H, *et al.* (2007) Fourteen polymorphic microsatellite loci characterized in the house sparrow *Passer domesticus* (Passeridae, Aves). *Molecular Ecology Notes*, **7**, 333-336.

Griffith SC, Stewart IRK, Dawson DA, Owens IPF, Burke T (1999) Contrasting levels of extra-pair paternity in mainland and island populations of the house sparrow (*Passer domesticus*): is there an 'island effect'? *Biological Journal of the Linnean Society*, **68**, 303-316.

Griffiths R, Double MC, Orr K, Dawson RJ (1998) A DNA test to sex most birds. *Molecular Ecology Notes*, **7**, 1071-1075.

Neumann K, Wetton JH (1996) Highly polymorphic microsatellites in the house sparrow *Passer domesticus*. *Molecular Ecology*, **5**, 307-309.

Richardson DS, Jury FL, Dawson DA, *et al.* (2000) Fifty Seychelles warbler (*Acrocephalus sechellensis*) microsatellite loci polymorphic in Sylviidae species and their cross-species amplification in other passerine birds. *Molecular Ecology*, **9**, 2226-2231.
